# Dietary BmKn2-7 Supplementation Modulates Intestinal Microbiota and Hepatopancreatic Responses and Enhances Resistance to *Vibrio parahaemolyticus* in *Litopenaeus vannamei*

**DOI:** 10.3390/metabo16100797

**Published:** 2026-10-21

**Authors:** Qiang Chen, Qi Wang, Qihang Liang, Beiping Tan, Shiwei Xie, Junming Deng, Wei Zhang, Hongyu Liu, Ling Zeng

**Affiliations:** 1 Laboratory of Aquatic Animal Nutrition and Feed, Fisheries College, Guangdong Ocean University, Zhanjiang 524088, China; 13226253418@126.com (Q.C.); 18409496835@163.com (Q.W.); yuanhl1002@163.com (Q.L.); bptan@126.com (B.T.); xiesw@gdou.edu.cn (S.X.); djunming@163.com (J.D.); 2 State Key Laboratory of Quality Research in Chinese Medicine, Macau University of Science and Technology, Macau 999078, China; wzhang@must.edu.mo; 3 School of Chemistry and Chemical Engineering, Lingnan Normal University, Zhanjiang 524048, China

**Keywords:** antimicrobial peptide, *Litopenaeus vannamei*, *Vibrio parahaemolyticus*, florfenicol

## Abstract

**Background:** Global expansion of *Litopenaeus vannamei* aquaculture has intensified the reliance on antibiotics for disease control, raising concerns about hepatotoxicity, microbiota dysbiosis and antimicrobial resistance. Synthesized scorpion venom-derived antimicrobial peptide BmKn2-7 is a promising alternative, but its efficacy and mechanisms as a dietary additive in shrimp remain unclear. **Methods:** A 56-day feeding trial was conducted with six diets: a basal diet (FM), four diets supplemented with BmKn2-7 (0.8, 1.6, 2.4 and 3.2 mg/kg), and a florfenicol diet (FL, 3.2 mg/kg). **Results:** Growth performance was unaffected by any treatment. However, FL group significantly reduced condition factor and hepatosomatic index and markedly increased serum ALT and AST. The A2.4 diet increased whole-body crude lipid contents and significantly elevated serum ACP, AKP, HDL-C, and TG. 16S rRNA gene sequencing showed that intestinal microbial diversity indices were numerically highest in the A2.4 group, although pairwise differences were not statistically significant. A2.4 was also associated with reduced *Vibrio* abundance and enrichment of genera such as *Acinetobacter*, while FL induced pronounced community shifts. Hepatopancreatic transcriptomics and pathway analysis indicated that A2.4 was associated with shifts in amino acid, organic acid, and carbohydrate metabolism, together with enhanced endoplasmic reticulum protein processing, whereas FL was mainly associated with reduced V-ATPase-related proton transport. Key BmKn2-7 responsive genes were positively correlated with phosphatase activities and lipid parameters, and *Hsp90b1* was negatively correlated with ALT and AST. Following *V. parahaemolyticus* challenge, A2.4 showed the lowest cumulative mortality, significantly outperforming FL and high-dose BmKn2-7. **Conclusions:** Dietary supplementation with BmKn2-7 at 2.4 mg/kg improves intestinal microbial homeostasis, metabolic and proteostatic functions, and resistance to *V. parahaemolyticus*. These findings support the further evaluation of BmKn2-7 as a potential feed additive for reducing antibiotic dependence in shrimp aquaculture.

## 1. Introduction

Global farmed marine shrimp production surged by 86% over the last decade, exceeding 6.5 million tons valued at nearly USD 40 billion in 2019 [1]. This growth has occurred primarily in East and Southeast Asia and Latin America, with *Litopenaeus vannamei* accounting for 83.1% of total production [2]. Rising global demand has driven the intensification of shrimp aquaculture, leading to the widespread adoption of high-density farming systems [3]. However, intensive production systems are frequently associated with recurrent disease outbreaks, which can cause substantial economic losses and raise food-safety concerns [4]. Conventional disease management frequently relies on antibiotics like florfenicol and norfloxacin [5]. Nevertheless, extensive research has highlighted significant safety and environmental issues associated with antibiotic overuse [6,7]. Reported consequences include adverse physiological effects in shrimp, the accumulation of antibiotic residues in the food chain, and the dissemination of antibiotic resistance genes (ARGs) [8]. Consequently, strategies to minimize antibiotic dependence in shrimp farming are actively being pursued. Promising alternatives under investigation include probiotics, immunostimulants, bacteriophages, organic acids, plant-derived compounds, and antimicrobial peptides (AMPs) [9]. Among these alternatives, AMPs have attracted particular attention because of their broad-spectrum antimicrobial activity and potentially favorable environmental profile [10].

AMPs are naturally occurring components of the innate immune system that are widely distributed across diverse taxa and exhibit potent activity against a broad range of pathogens; some AMPs also possess antitumor activity [11]. Compared with probiotics, which often require stringent storage and handling conditions, AMPs may offer greater stability in aquafeed formulations and can exert rapid bactericidal activity [12]. Their mechanisms of action encompass membrane disruption, intracellular targeting, and modulation of host immune responses [13]. Research consistently demonstrates the efficacy of dietary AMP supplementation in aquatic species. For example, supplementation at levels of 400–800 mg/kg AMPs improved intestinal morphology, boosted gut microbiota diversity, and promoted beneficial probiotic abundance in grass carp (*Ctenopharyngodon idellus*) [14]. Similarly, supplementation with the AMP CM11 at 50 μg/g significantly upregulated the expression of antioxidant- and immune-related genes in the liver of zebrafish (*Danio rerio*) [15]. Dietary supplementation with AMPs at 400–800 mg/kg also enhanced resistance to *Aeromonas hydrophila* infection in Pengze crucian carp (*Carassius auratus* var. *Pengze*) [16]. In vitro studies showed that an AMP derived from fermented soybean exerted bactericidal activity against *Vibrio parahaemolyticus* by disrupting cell membrane integrity. Subsequent in vivo experiments demonstrated that dietary supplementation with this AMP at 62.5 μg/g significantly increased the survival of *L. vannamei* following challenge with *V. parahaemolyticus* [17]. Furthermore, AMP supplementation at 62.5 μg/g modulated the shrimp gut microbiota, enriching beneficial bacterial communities while suppressing pathogenic *Vibrio* and *Flavobacterium* populations, concurrently enhancing microbial diversity [18]. In another study, dietary inclusion of AMPs at 0.4% significantly improved growth performance, enhanced antioxidant capacity, and promoted innate immune responses in *L. vannamei* [19].

Among AMPs, scorpion venom-derived peptides display considerable structural diversity and antimicrobial activity and have attracted increasing attention as potential candidates for aquaculture applications [20,21]. Representative scorpion venom-derived AMPs include hadrurin, IsCT, scorpine and StCT1, which have been reported to exhibit diverse biological activities [22,23,24,25]. BmKn2-7, an engineered derivative of the *Mesobuthus martensii* Karsch peptide BmKn2, offers improved antibacterial properties and reduced hemolytic toxicity [26]. However, the physiological effects and safety of dietary BmKn2-7, as well as the mechanisms underlying its activity, have not been characterized in shrimp. It also remains unclear whether dietary BmKn2-7 confers physiological benefits beyond its direct antibacterial effects. Because dietary AMPs have been reported to modulate the gut microbiota, metabolism and immune responses in other aquatic species, determining whether BmKn2-7 elicits comparable effects is important for evaluating its potential as a feed additive. Furthermore, although florfenicol is widely used as a conventional antibiotic in shrimp farming, increasing evidence indicates risks of hepatotoxicity, microbiota disruption and reduced host resilience [27]. Moreover, BmKn2-7 has not been directly compared with florfenicol in shrimp, leaving its relative physiological effects and potential as an antibiotic-sparing feed additive unresolved. A further question is whether AMP-induced shifts in the intestinal microbiota are associated with hepatopancreatic metabolic and proteostatic processes, including AMPK signaling and endoplasmic reticulum proteostasis, and whether these coordinated responses contribute to host health and disease resistance. To address these questions, we conducted an eight-week feeding trial in *L. vannamei* using graded dietary levels of BmKn2-7 and a florfenicol control, integrating analyses of growth, serum biochemistry, whole-body composition, gut microbiota and hepatopancreatic transcriptomics, followed by a *V. parahaemolyticus* challenge. By integrating phenotypic, microbial, and molecular evidence, this study aimed to elucidate the effects of dietary BmKn2-7 supplementation on growth performance, intestinal microbiota, and immune responses in *L. vannamei*. This study provides a basis for further evaluating BmKn2-7 as a potential antibiotic-sparing feed additive in shrimp aquaculture.

## 2. Materials and Methods

### 2.1. Diet Preparation and Experimental Design

The BmKn2-7 peptide (FIKRIARLLRKIF-NH_2_) was commercially synthesized by solid-phase peptide synthesis (SPPS) with C-terminal amidation by Sangon Biotech Co., Ltd. (Shanghai, China). Peptide purity (>98%) was determined by reversed-phase high-performance liquid chromatography (RP-HPLC) using a C18 analytical column (4.6 × 250 mm, 5 μm) with UV detection at 220 nm. Chromatographic separation was performed at a flow rate of 1.0 mL/min using a linear acetonitrile gradient from 10% to 90% over 30 min in the presence of 0.1% trifluoroacetic acid (TFA). Lyophilized BmKn2-7 was stored at −80 °C until experimental use. Florfenicol (purity ≥98%) was procured from Shanghai Aladdin Bio-Chem Technology Co., Ltd. (Shanghai, China) and stored at −20 °C prior to use. The experimental diets contained the following components: fish meal (25%), soybean meal (11%), peanut meal (11%), corn gluten meal (13%), and shrimp shell meal (5%) as major protein sources; fish oil (2%) and soy lecithin (2.5%) served as lipid sources, supplemented with wheat flour (25%) as a carbohydrate base. Graded levels of BmKn2-7 were added to the basal diet: 0 mg/kg (FM), 0.8 mg/kg (Diet A0.8), 1.6 mg/kg (Diet A1.6), 2.4 mg/kg (Diet A2.4), and 3.2 mg/kg (Diet A3.2). The dietary inclusion levels of BmKn2-7 (0.8, 1.6, 2.4, and 3.2 mg/kg diet) were selected based on the potent antimicrobial activity of this synthetic peptide. Previous studies demonstrated that BmKn2-7 exhibited strong inhibitory activity against *V. parahaemolyticus* with a minimum inhibitory concentration (MIC) of 125 μg/mL [28]. An additional diet supplemented with 3.2 mg/kg florfenicol served as the antibiotic control (FL). The florfenicol supplementation level was selected based on previous studies [29]. All feed ingredients were ground to pass through an 80-mesh sieve, accurately weighed according to the formulation, and thoroughly mixed using a stepwise dilution procedure. The resulting mixture was extruded into pellets with diameters of 1.0 and 1.5 mm using an F-75 twin-screw extruder (South China University of Technology, Guangzhou, China). After extrusion, the pellets were conditioned at 60 °C for 30 min and subsequently air-dried at room temperature for 48 h. The prepared diets were vacuum-sealed and stored at −20 °C until use. The ingredient composition and proximate composition of the experimental diets are presented in Table 1.

### 2.2. Culture Management

The feeding trial was conducted at the Marine Biology Research Base of Guangdong Ocean University. Juvenile *L. vannamei* obtained from Zhanjiang Zhonglian Aquaculture Co., Ltd. (Zhanjiang, China), were acclimated in outdoor concrete tanks for four weeks before the feeding trial. A total of 720 apparently healthy shrimp of similar size (initial body weight, 0.26 ± 0.00 g) were randomly assigned to six dietary treatments, with three replicate tanks per treatment and 40 shrimp per tank. During the eight-week feeding trial, shrimp were fed four times daily at 07:00, 11:00, 17:00 and 21:00. The volume of each culture tank was 0.3 m^3^. Approximately 50% of the tank water was exchanged each morning. During the feeding trial, water quality parameters were maintained as follows: temperature 26–28 °C, dissolved oxygen ≥ 6 mg/L, and nitrite concentration ≤ 0.5 mg/L.

### 2.3. Sample Collection

At the end of the 56-day feeding trial, shrimp were fasted for 24 h before sampling. The surviving shrimp in each replicate tank were counted and weighed. Ten shrimp were randomly selected from each replicate tank for hemolymph collection. Hemolymph was withdrawn from the pericardial sinus using sterile syringes. Hemolymph samples were maintained at 4 °C for 12 h, then centrifuged at 3500× *g* for 10 min to obtain serum. Serum aliquots were stored at −80 °C for enzymatic activity assays. Meanwhile, intestinal tissues were dissected from three shrimp per tank, snap-frozen in liquid nitrogen, and then used for microbial community analysis. Three additional whole shrimp from each tank were stored at −20 °C for subsequent whole-body proximate composition analysis. Two shrimp were randomly sampled from each tank, and their hepatopancreatic tissues were collected, snap-frozen in liquid nitrogen, and subjected to transcriptome sequencing.

### 2.4. Proximate Composition Analysis

The proximate composition of feed ingredients, experimental diets and whole-body shrimp samples was determined using standardized analytical methods. After homogenization, moisture content was determined by oven-drying at 105 °C to constant weight in accordance with GB/T 6435-2014. Crude protein content was determined using a Primacs SN Dumas nitrogen analyzer (Skalar, The Netherlands). Crude lipid content was determined by Soxhlet extraction in accordance with the applicable GB/T 6433 standard. Crude ash content was determined by incineration in a muffle furnace in accordance with the applicable GB/T 6438 standard. All analytical procedures incorporated standardized pretreatment protocols to ensure measurement accuracy through rigorous quality control measures.

### 2.5. Serum Biochemical Analysis

Serum biochemical parameters were determined using a full-wavelength microplate reader (Thermo Fisher Scientific, Waltham, MA, USA). The measured indices included: triglyceride (TG, A110-1-1), total cholesterol (T-CHO, A111-1-1), acid phosphatase (ACP, A060-2-2), alkaline phosphatase (AKP, A059-2-2), low-density lipoprotein (LDL, A113-1-1), high-density lipoprotein (HDL, A112-1-1), aspartate aminotransferase (AST, C010-2-1), and alanine aminotransferase (ALT, C009-2-1). All assays were performed using commercial kits (Nanjing Jiancheng Bioengineering Institute, Nanjing, China) strictly following manufacturer protocols.

### 2.6. Intestinal Microbial Community Analysis

Intestinal microbial communities were characterized by 16S rRNA gene amplicon sequencing performed by Gene Denovo Biotechnology Co., Ltd. (Guangzhou, China). Microbial genomic DNA was extracted from intestinal samples using the HiPure Soil DNA Extraction Kit (Guangzhou Maihe Biotechnology Co., Ltd., Guangzhou, China) according to the manufacturer’s instructions. The V3-V4 hypervariable region of the bacterial 16S rRNA gene was amplified using the universal primers 341F (5′-CCTACGGGNGGCWGCAG-3′) and 806R (5′-GGACTACHVGGGTWTCTAAT-3′). PCR amplification was performed under the following conditions: initial denaturation at 95 °C for 5 min; 30 cycles of denaturation at 95 °C for 1 min, annealing at 60 °C for 1 min and extension at 72 °C for 1 min; followed by a final extension at 72 °C for 7 min. Each 50-µL PCR mixture contained 10 µL of 5× Q5 Reaction Buffer, 10 µL of 5× Q5 High GC Enhancer, 1.5 µL of 2.5 mM dNTPs, 1.5 µL of each primer (10 µM), 0.2 µL of Q5 High-Fidelity DNA Polymerase and 50 ng of template DNA. PCR reagents were obtained from New England Biolabs (Ipswich, MA, USA). PCR products were evaluated by 2% agarose gel electrophoresis and purified using AMPure XP beads (Beckman Coulter, CA, USA) according to the manufacturer’s instructions. Sequencing libraries were prepared using the Illumina DNA Prep Kit (Illumina, CA, USA) according to the manufacturer’s instructions and quantified using an ABI StepOnePlus Real-Time PCR System (Life Technologies, Foster City, CA, USA). Paired-end sequencing (2 × 250 bp) was performed on an Illumina NovaSeq 6000 platform.

### 2.7. Transcriptomic Analysis of Hepatopancreas

Hepatopancreas samples from the FM, A2.4, and FL groups were selected for transcriptome sequencing. Hepatopancreas samples were collected from two shrimp per tank and pooled as one biological replicate (stored at −80 °C). Therefore, three biological replicates per treatment group were obtained for RNA extraction and subsequent transcriptomic analysis. The sequencing workflow proceeded as follows: Polyadenylated mRNA was enriched using oligo-dT magnetic beads (mRNA Capture Beads). Following purification, mRNA was fragmented at elevated temperature (94 °C for 5 min). First-strand cDNA synthesis was performed using fragmented mRNA as template with reverse transcriptase (SuperScript™ IV, Thermo Fisher). Following second-strand cDNA synthesis, the double-stranded cDNA underwent end repair and 3′-adenylation. Illumina-compatible adapters were ligated to adenylated fragments, followed by size selection (350–450 bp) using Hieff NGS^®^ DNA Selection Beads. The adapter-ligated libraries were amplified by 12 cycles of PCR. The resulting libraries were sequenced on an Illumina NovaSeq X Plus platform. Raw sequencing data underwent quality control via Fastp (v0.23.2) with adapter trimming and quality filtering (Q30 > 90%). High-quality clean reads were aligned to the *L. vannamei* reference genome assembly (GCF_015228065.1) using HISAT2 (v2.2.1). Gene-level read counts were quantified using featureCounts (v2.0.3) to generate a raw count matrix. Principal component analysis (PCA) was implemented in R (v4.3.1) using the prcomp function. Differentially expressed genes (DEGs) were identified using edgeR (v3.42.4), with thresholds of |log_2_ fold change| > 1 and false discovery rate (FDR) < 0.05. Functional enrichment of DEGs was analyzed through Gene Ontology (GO) and Kyoto Encyclopedia of Genes and Genomes (KEGG) databases using clusterProfiler (v4.8.1).

### 2.8. Validation of RNA-Seq Data by qRT-PCR

The same RNA samples used for RNA-seq were subjected to reverse transcription quantitative PCR (RT-qPCR) to validate the RNA-seq results. A total of 24 DEGs were selected for validation based on the RNA-seq results. The primer sequences used for RT-qPCR are listed in Table 2 and were synthesized by Shenggong Biological Engineering Co., Ltd. (Shanghai, China). RT-qPCR reaction mixtures were prepared according to the manufacturer’s instructions for the SYBR^®^ Premix Ex Taq™ kit (Takara, Kusatsu, Japan), and amplification and fluorescence detection were performed using an Applied Biosystems 7500 Real-Time PCR System (Life Technologies, Foster City, CA, USA). All experimental operations were carried out in strict accordance with the instructions. Thermal cycling was performed at 95 °C for 2 min, followed by 40 cycles at 95 °C for 10 s and 72 °C for 20 s. In addition, all reactions were operated in triplicate. The gene expression was calculated using the 2^−ΔΔCT^ method, described by Wang et al. [30].

### 2.9. V. parahaemolyticus Challenge Test

Following the feeding trial, a bacterial challenge was conducted using *V. parahaemolyticus* (strain VpGX01, accession no. GDMCC 1.3687) supplied by the Guangdong Provincial Key Laboratory of Aquatic Disease Control and Healthy Culture (Zhanjiang). Thirty apparently healthy shrimp from each dietary treatment (10 shrimp per replicate tank) were subjected to the challenge test. Each shrimp was intramuscularly injected into the third abdominal segment with 100 μL of a bacterial suspension containing 1 × 10^7^ CFU mL^−1^ (LD50). The challenge concentration was selected based on preliminary challenge experiments. Mortality was recorded at 4-h intervals for 52 h after challenge, and cumulative mortality rate was calculated for each replicate tank.

### 2.10. Statistical Analysis

Data are presented as the mean ± standard error of the mean (SEM, *n* = 3), and statistical analyses were performed using IBM SPSS Statistics 23.0. Three independent replicate tanks were used for each group. SEM values were calculated based on the three biological replicates within each treatment group. Differences among dietary treatments were assessed by one-way analysis of variance (ANOVA), followed by Duncan’s multiple range test. Intestinal flora and transcriptome analyses were performed on the Omicsmart dynamic online analysis platform (http://www.omicsmart.com). Differences in α-diversity indices among groups were assessed using the Kruskal–Wallis test, followed by Dunn’s post hoc test with Bonferroni correction for multiple comparisons. A *p* value < 0.05 was considered statistically significant. Challenge test cumulative survival was analyzed on Graphpad prism software (version 10.1.2) using the Gehan Breslow-Wilcoxon method.

## 3. Results

### 3.1. Growth Performance and Morphology Indices

The growth performance and morphology indices in the different dietary treatments are presented in Table 3. IBW showed no significant differences across all groups (*p* > 0.05). FBW ranged from 13.42 ± 0.43 g (A1.6) to 13.97 ± 0.23 g (A3.2), with no statistical variations observed (*p* > 0.05). Similarly, WGR, SGR, FCR, and SR did not differ significantly among treatments (*p* > 0.05). Notably, significant differences were detected in CF and HSI. The FL group exhibited the lowest CF value (0.82 ± 0.00%) compared to FM (0.98 ± 0.04%) and A3.2 (1.00 ± 0.04%) groups (*p* < 0.05). Similarly, HSI in the FL group (3.89 ± 0.23%) was markedly reduced relative to other groups (*p* < 0.05). The results indicated that neither BmKn2-7 nor florfenicol exerted significant effects on shrimp growth parameters (*p* > 0.05). However, florfenicol significantly reduced the HSI (*p* < 0.05), suggesting potential hepatopancreatic atrophy induced by this treatment.

### 3.2. Whole-Body Composition

The whole-body proximate composition of shrimp under different dietary treatments is presented in Table 4. Moisture content did not differ significantly among dietary treatments (*p* > 0.05). Crude protein content was notably lower in the A2.4 group than in the FM group (*p* < 0.05), but did not differ notably between the other groups and the FM group (*p* > 0.05). For crude lipid, the A1.6 group displayed the highest value (*p* < 0.05), followed by A2.4 and A0.8, all markedly exceeding the FM control. In contrast, crude lipid in the A3.2 group and FL group was markedly lower than A1.6 and A2.4 groups (*p* < 0.05). The crude ash content was significantly higher in the A0.8 group than in the FM and FL groups (*p* < 0.05). These results suggest that dietary supplementation with BmKn2-7 at 1.6–2.4 mg/kg enhanced lipid deposition, while higher concentrations (3.2 mg/kg) or florfenicol attenuated this effect.

### 3.3. Serum Biochemical Indices

**Figure 1:**
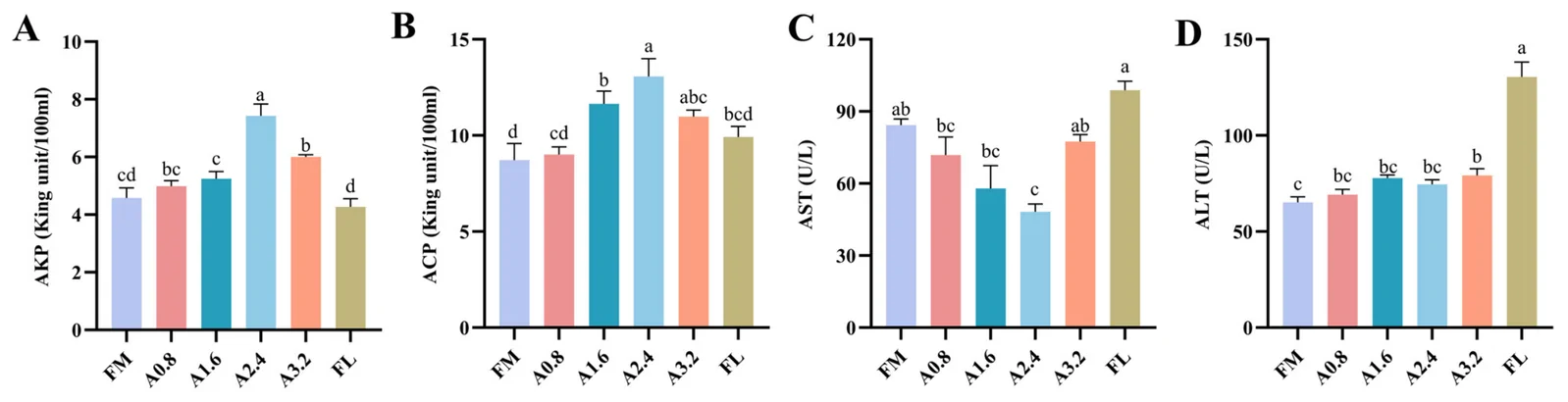
Effects of BmKn2-7 on serum biochemical indices of *L. vannamei*. (**A**): AKP activity; (**B**): ACP activity; (**C**): AST activity; (**D**): ALT activity; Data are presented as mean ± SEM (*n* = 3). Different letter superscripts indicate significant differences (*p* < 0.05).

As shown in Figure 1, serum enzyme activities differed significantly among dietary treatments (*p* < 0.05). ACP activity was markedly elevated in the A2.4 group compared to FM and A0.8 (*p* < 0.05), while A1.6 and A3.2 also exceeded FM (*p* < 0.05). AKP activity peaked in A2.4, surpassing all other groups, with A3.2 notably higher than FM and FL (*p* < 0.05). ALT activity was markedly increased in FL relative to all other groups (*p* < 0.05), whereas BmKn2-7 groups (A0.8-A2.4) showed no significant difference from FM (*p* > 0.05), except for A3.2 group (*p* < 0.05). AST activity was significantly higher in the FL group than in the A2.4 group (*p* < 0.05), but did not differ significantly between the FL group and the FM or A3.2 groups (*p* > 0.05). The A2.4 group had the lowest AST activity and was significantly lower than all other groups (*p* < 0.05).

**Figure 2:**
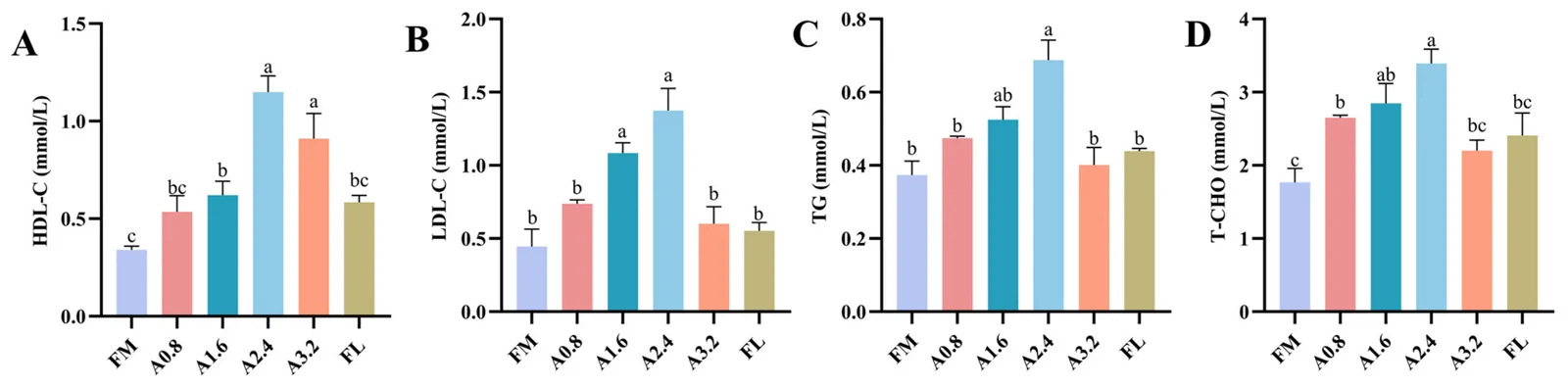
Effects of BmKn2-7 on serum biochemical indices of *L. vannamei*. (**A**): HDL-C concentrations; (**B**): LDL-C concentrations; (**C**): TG concentrations; (**D**): T-CHO concentrations. Data are presented as mean ± SEM (*n* = 3). Different letter superscripts indicate significant differences (*p* < 0.05).

Regarding serum lipid metabolism (Figure 2), HDL-C concentrations were considerably higher in A2.4 and A3.2 versus FM and FL (*p* < 0.05). LDL-C concentrations were elevated in A1.6 and A2.4 compared to FM, A0.8, A3.2, and FL (*p* < 0.05). T-CHO concentrations were highest in the A2.4 group and differed notably from all other groups except the A1.6 group (*p* < 0.05). TG concentration was highest in A2.4, notably greater than FM, A0.8, A3.2, and FL (*p* < 0.05), though A1.6 did not differ notably from FM (*p* > 0.05). Overall, dietary BmKn2-7 supplementation, particularly at 2.4 mg/kg, altered serum lipid-related parameters, whereas the effects were less pronounced at 3.2 mg/kg. Florfenicol supplementation was associated with increased serum ALT and AST activities.

### 3.4. Intestinal Microbiota Analysis

#### 3.4.1. Diversity Analysis of the Intestinal Microbiota

**Figure 3:**
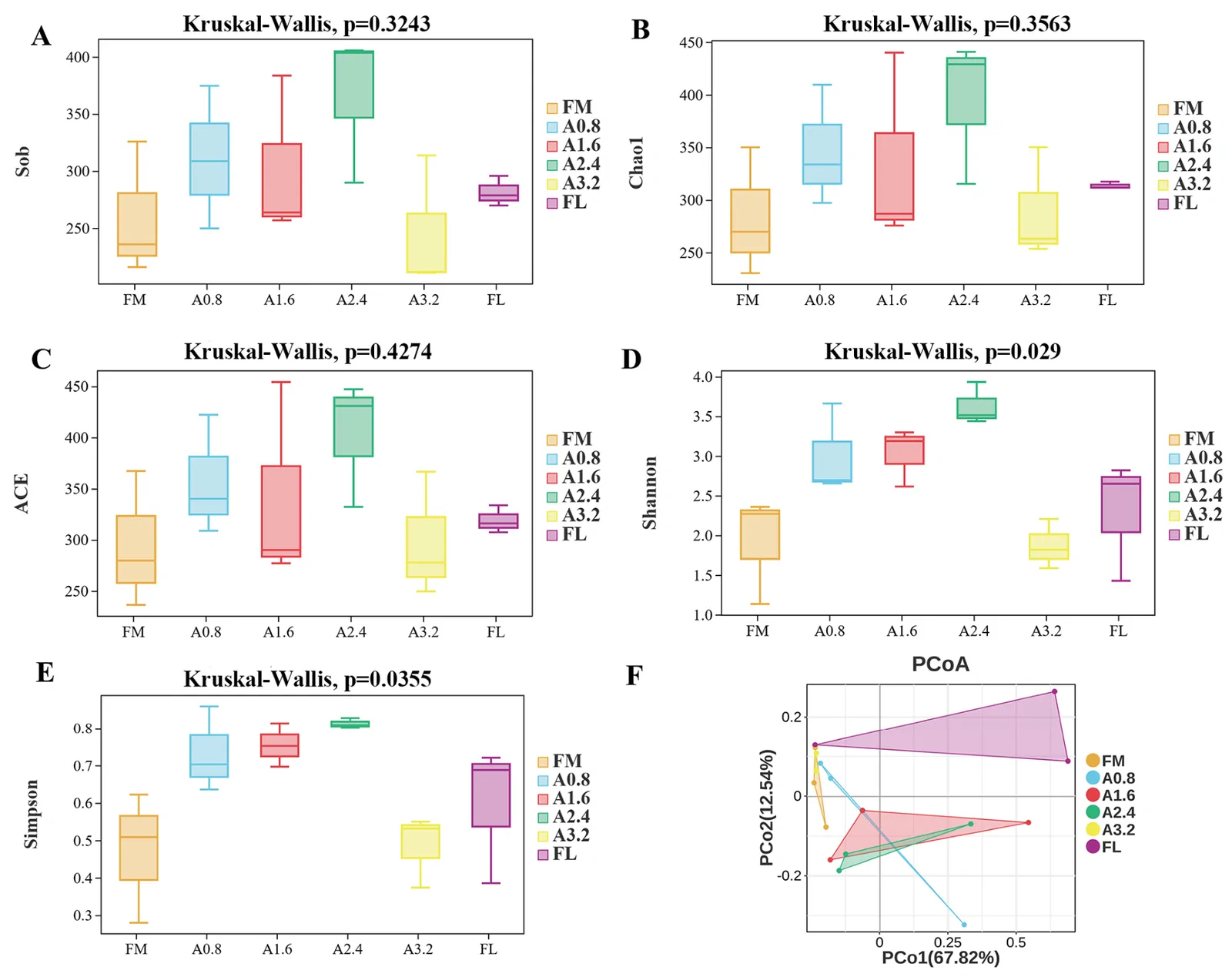
Effects of BmKn2-7 on the intestinal microbiota diversity in *L. vannamei*. (**A**): Sob index; (**B**): Chao1 index; (**C**): ACE index; (**D**): Shannon index; (**E**): Simpson index; (**F**): principal co-ordinates analysis. Statistical significance was evaluated using the Kruskal–Wallis test followed by Dunn’s post hoc test with Bonferroni correction.

Differences in alpha diversity indices of the intestinal microbiota were analyzed using the Kruskal–Wallis test, and the corresponding box plots are presented in Figure 3. The Good’s coverage index was above 99.9%, indicating that the sequencing quality of the samples met the requirements for analysis. Sob, Chao1, ACE, Shannon and Simpson indexes were all highest in the A2.4 group. Significant differences in the Shannon and Simpson indices were observed among the groups (*p* < 0.05). However, no significant pairwise differences were detected after Dunn’s post hoc test with Bonferroni correction. β-diversity analysis of the intestinal microbiota at the genus level was performed based on Bray–Curtis distances. According to principal coordinate analysis (PCoA), the distance between FL and other groups were relatively large, suggesting great differences in the structure of the intestinal microbiota between FL and other groups (Figure 3F).

#### 3.4.2. Analysis of Species Composition

**Figure 4:**
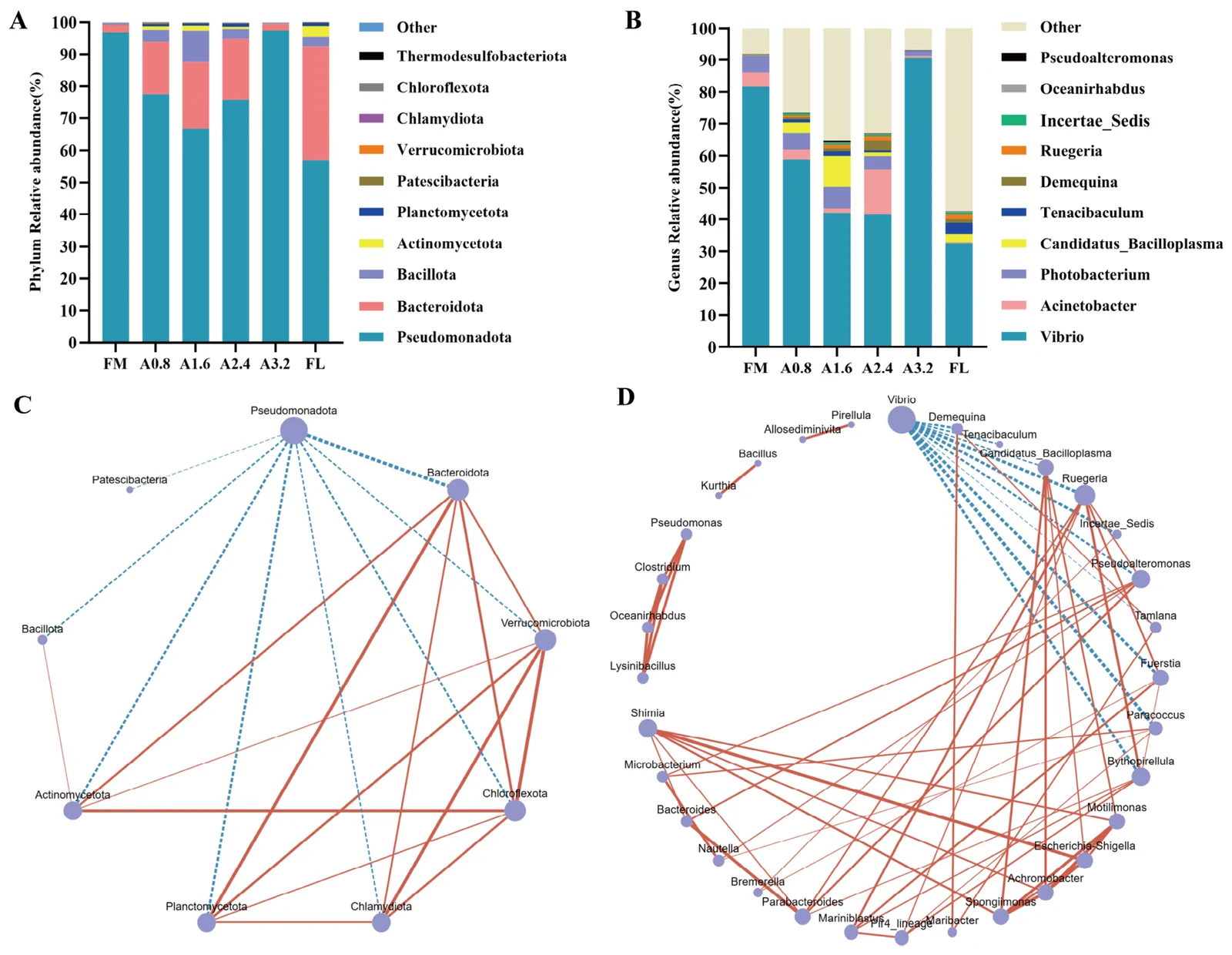
Analysis of the species composition of the intestinal microbiota. (**A**): Relative abundances of the 10 most abundant phyla. (**B**): Relative abundances of the 10 most abundant genera. (**C**): Species correlation network diagram based on Pearson correlation analysis (Phylum level, |r| > 0.5, *p* < 0.05). Red lines indicate positive correlation. Blue dashed lines indicate negative correlations. Line thickness is determined by the correlation coefficient. (**D**): Species correlation network diagram based on Pearson correlation analysis (genus level, |r| > 0.5, *p* < 0.05).

Microbial community analysis revealed that Pseudomonadota dominated all groups at the phylum level (relative abundance: 56.95–97.55%) (Figure 4A), followed by Bacteroidota (2.01–35.46%) and Bacillota (0.27–9.91%). The A3.2 (97.55%) and FM (96.88%) groups exhibited peak abundance of Pseudomonadota, while the FL group demonstrated a distinct community structure with significantly elevated Bacteroidota (35.46%). At the genus level (Figure 4B), *Vibrio* was the dominant genus in all groups except FL, with relative abundances ranging from 41.63% to 90.73%. Acinetobacter showed a relatively high abundance in the A2.4 group (13.94%). The FL group showed reduced *Vibrio* abundance (32.40%), accompanied by higher proportions of *Demequina* (3.34%) and *Tenacibaculum* (1.16%), alongside substantial unclassified microorganisms (55.33%), indicating unique microbial compositional characteristics, consistent with the beta-diversity results. Comparative analysis showed that the relative abundance of *Vibrio* was higher in the FM group (81.64%) than in the A1.6 (41.99%), A2.4 (41.63%) and FL (32.40%) groups. In contrast, the FL group showed lower *Vibrio* abundance than the A0.8 (58.81%) and A3.2 (90.73%) groups.

Correlation network analysis was performed to explore co-occurrence patterns among dominant taxa (Figure 4C,D). At the phylum level, Pseudomonadota showed a negative correlation with several other phyla, including Bacteroidota, whereas Bacteroidota displayed positive associations with multiple low-abundance phyla. At the genus level, *Vibrio* exhibited negative correlations with several genera, while *Acinetobacter* showed positive associations with a subset of taxa. These correlations indicate shifts in microbial co-occurrence structure among dietary treatments, but the functional implications of these associations remain to be verified.

#### 3.4.3. Indicator Species Analysis

**Figure 5:**
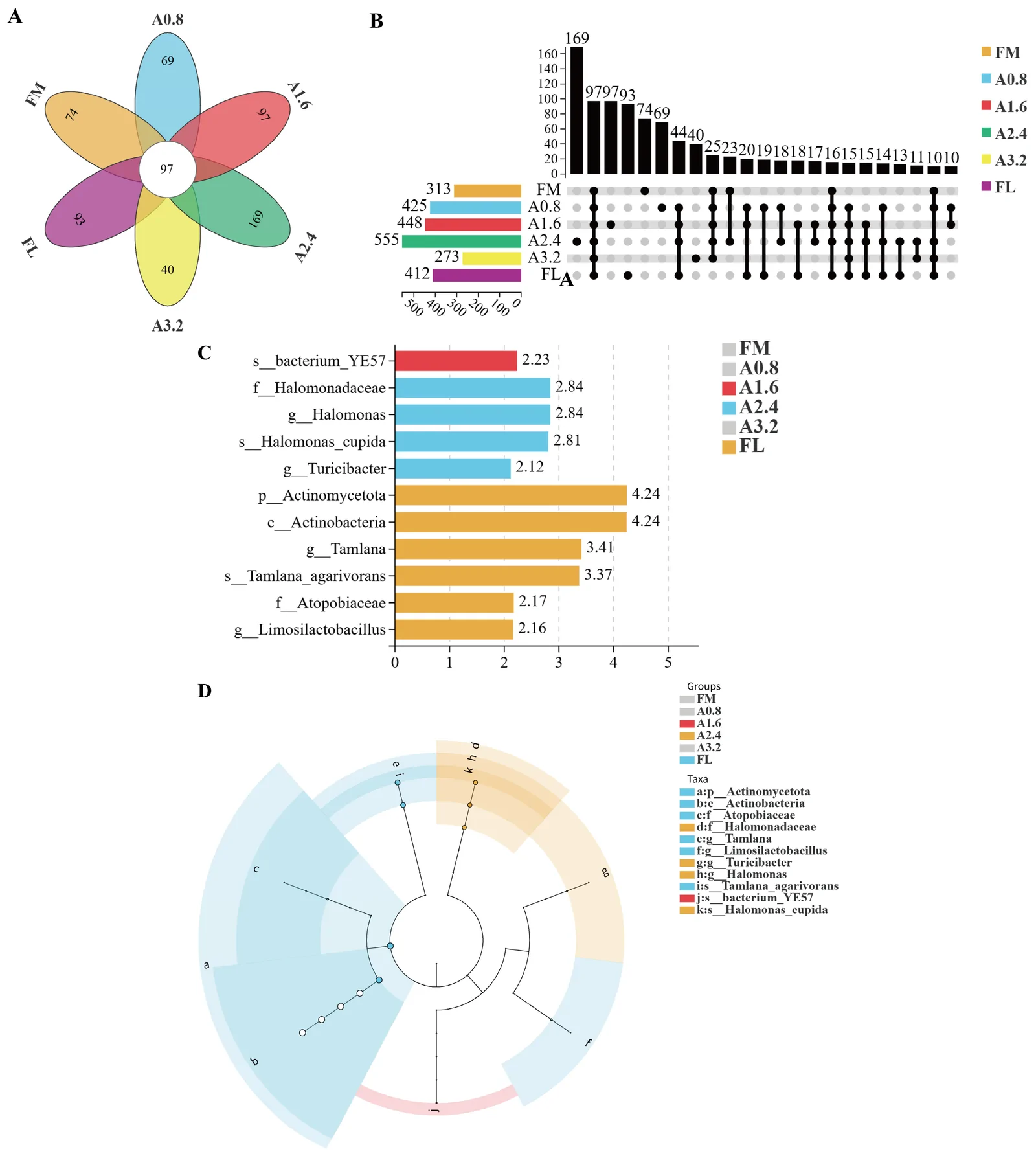
Indicator species analysis of the intestinal microbiota. (**A**,**B**): Venn and upset diagrams based on OTU levels. (**C**,**D**): Intestinal microbiota Linear Discriminant Analysis (LDA) Effect Size (LEfSe) analysis (LDA > 2, *p* < 0.05). (**C**): Histogram of LDA distribution. (**D**): Cladogram based on the LEfSe analysis.

The Venn diagram showed 97 shared OTUs among the six dietary treatments (Figure 5A). As for the unique OTUs for each group, there were 74 (FM), 69 (A0.8), 97 (A1.6), 169 (A2.4), 40 (A3.2), 93 (FL), respectively. Next, the Linear discriminant analysis effect size (LEfSe) analysis method was employed (Figure 5C), coupled with LDA scores, to identify differentially abundant species between groups. A total of 1, 4 and 6 discriminatory taxa were identified in the A1.6, A2.4 and FL groups, respectively. The FL group demonstrated significant enrichment of Actinomycetota at higher taxonomic ranks, concurrently featuring Bacteroidota-affiliated genera including *Tamlana* and *Limosilactobacillus*. Conversely, the A2.4 group exhibited pronounced enrichment of Halomonadaceae-associated taxa, particularly the genus *Halomonas*. Exclusive enrichment of bacterium_YE57 characterized the A1.6 microbiota. These biomarker profiles were consistent with the observed differences in microbial community composition, including the higher abundance of Bacteroidota in the FL group and increased Gammaproteobacteria representation in the A2.4 group.

#### 3.4.4. Mantel Test

**Figure 6:**
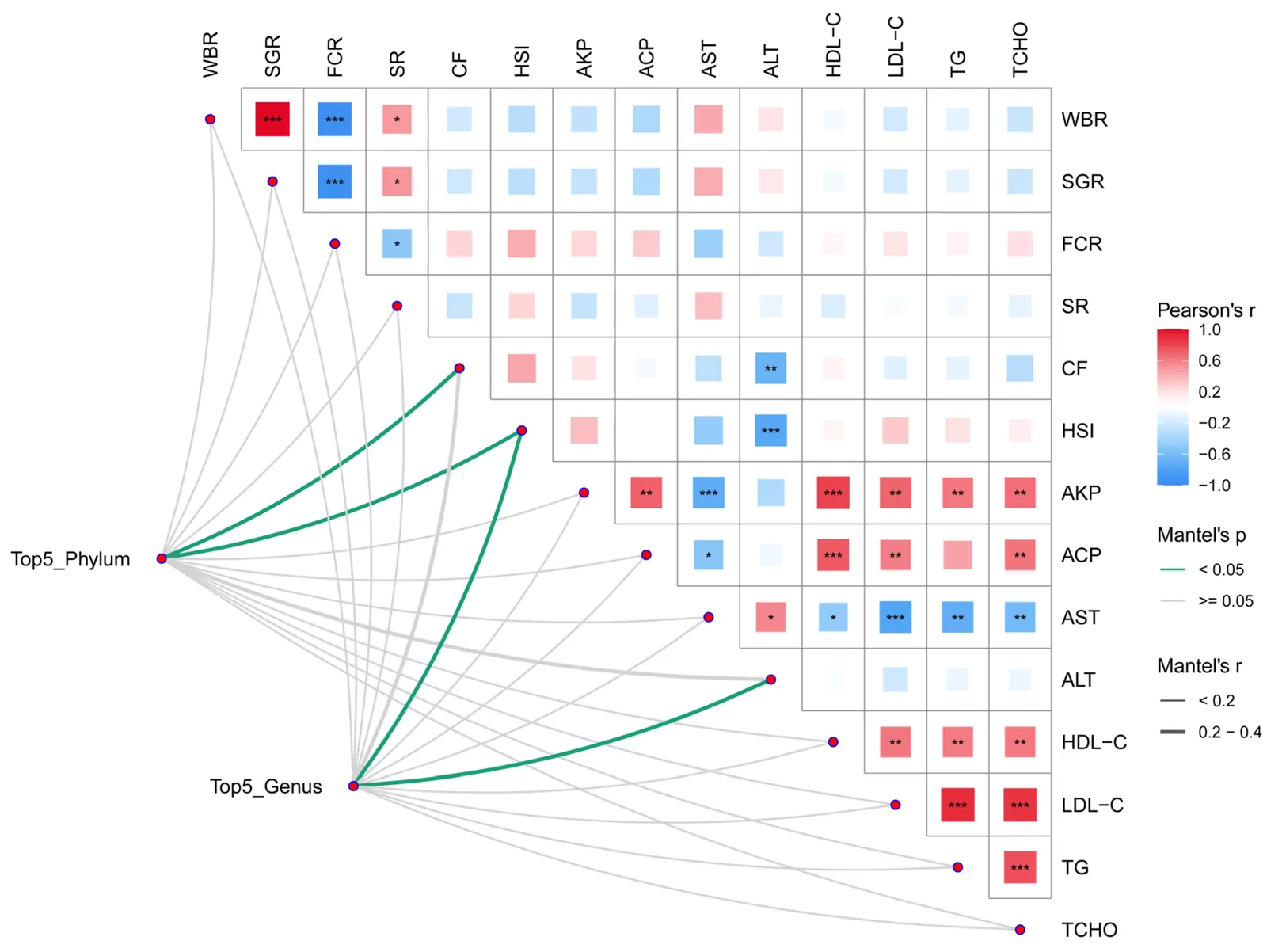
Pearson correlations among host traits and Mantel associations between gut microbiota composition and host phenotypic indices. Notes: The upper-right heatmap shows Pearson correlations among host traits (red, positive; blue, negative; * *p* < 0.05, ** *p* < 0.01, *** *p* < 0.001). Curved links indicate Mantel associations between microbiota composition (Bray–Curtis distances) and host phenotypes (Euclidean distances); green denotes *p* < 0.05 and gray denotes *p* ≥ 0.05, with line width proportional to Mantel’s r. “Top5_Phylum” and “Top5_Genus” represent the five most abundant phyla and genera, respectively.

Mantel tests were performed using Bray–Curtis distances calculated from the top five phylum and genus level abundance profiles and Euclidean distances of host physiological parameters. The results showed that microbiota compositional distances were significantly correlated with CF and HSI at the phylum level, and with HSI and ALT at the genus level (Figure 6). No significant Mantel correlations were detected for growth performance parameters or most serum biochemical indices.

### 3.5. Hepatopancreatic Transcriptome Analysis

#### 3.5.1. Sequencing Data Quality Assessment

To explore the molecular responses associated with the optimal BmKn2-7 supplementation level, transcriptomic analysis was performed using RNA sequencing (RNA-seq) in the FM, A2.4, and FL groups. The raw data (bp) produced by each treatment group was above 6 billion. The mean Q30 and Q20 values were 90.19% and 96.39%, respectively. Additionally, filtered reads exhibited a GC content of 49.15%. Based on the statistical results, it was found that transcriptome sequencing data had good sequencing quality, low error rate, high match rate, and high base quality.

#### 3.5.2. Assessment of Inter-Group Variation by Multivariate Analysis

**Figure 7:**
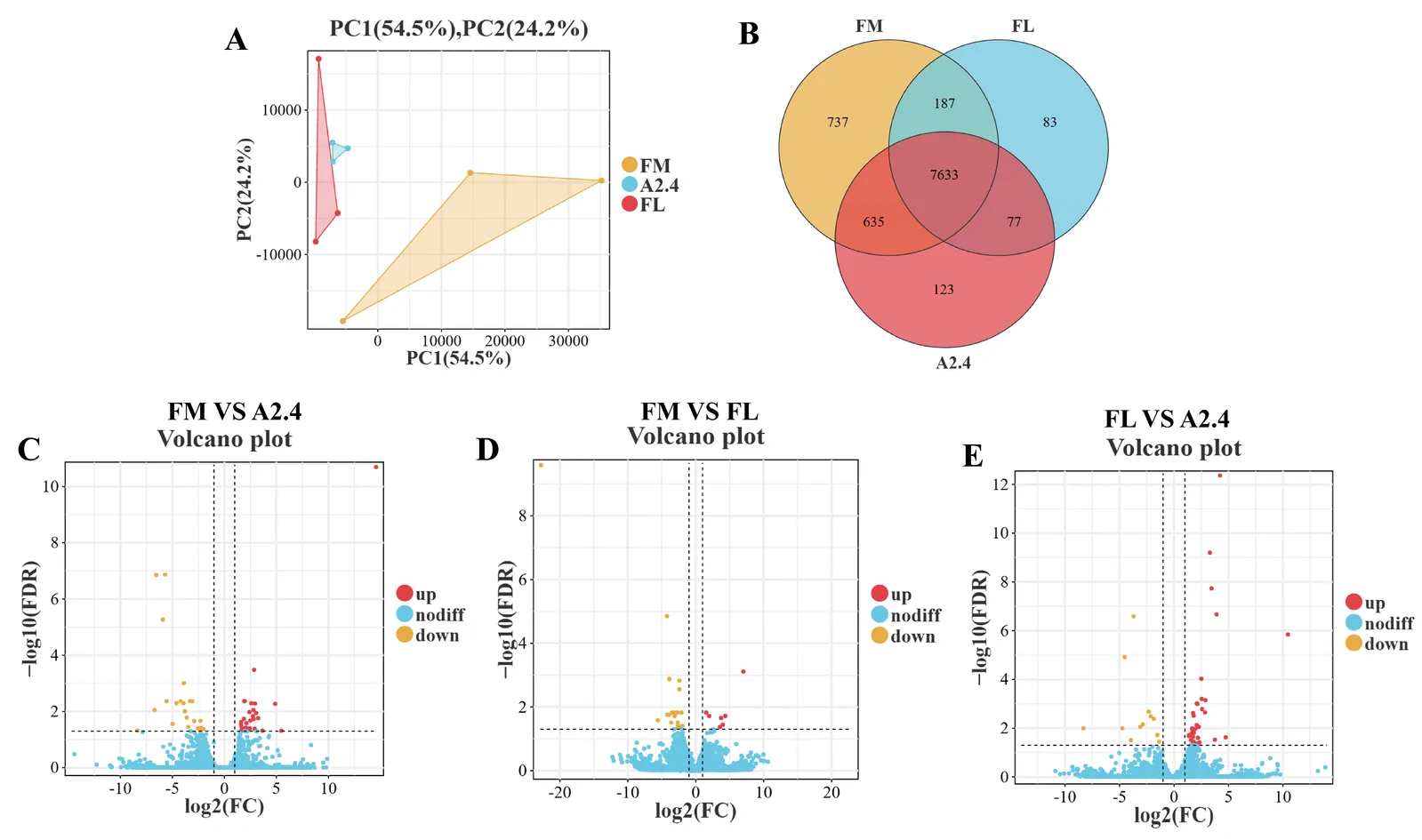
(**A**): PCA performed using three biological replicates per group (*n* = 3). (**B**): Venn diagram of expressed genes among the three groups. (**C**–**E**): Volcano map of differentially expressed genes. Differentially expressed genes were identified using EdgeR based on three biological replicates per group.

To evaluate global transcriptomic patterns, PCA was performed using variance-stabilized counts. The first two principal components explained 78.7% of total variance (PC1 = 54.5%; PC2 = 24.2%). The PCA plot showed close clustering of the FL and A2.4 groups, whereas both groups were separated from the FM group along the PC1-PC2 axes (Figure 7A). To compare the similarities and differences in gene expression profiles among the three groups, Venn analysis was performed on the genes detected in the three groups (Figure 7B). The results showed that a total of 7633 genes were shared among the three groups. Meanwhile, the FM, FL, and A2.4 groups contained 737, 83, and 123 unique genes, respectively.

#### 3.5.3. Analysis of Differentially Expressed Gene (DEG) Profiles

Differential expression analysis identified DEGs between the treatment conditions (Figure 7C–E). The FM vs. A2.4 comparison resulted in 42 downregulated and 43 upregulated genes (85 DEGs total). The FM vs. FL comparison resulted in 12 downregulated and 38 upregulated genes (50 DEGs total). The FL vs. A2.4 comparison resulted in 21 upregulated and 80 downregulated genes (101 DEGs total).

**Figure 8:**
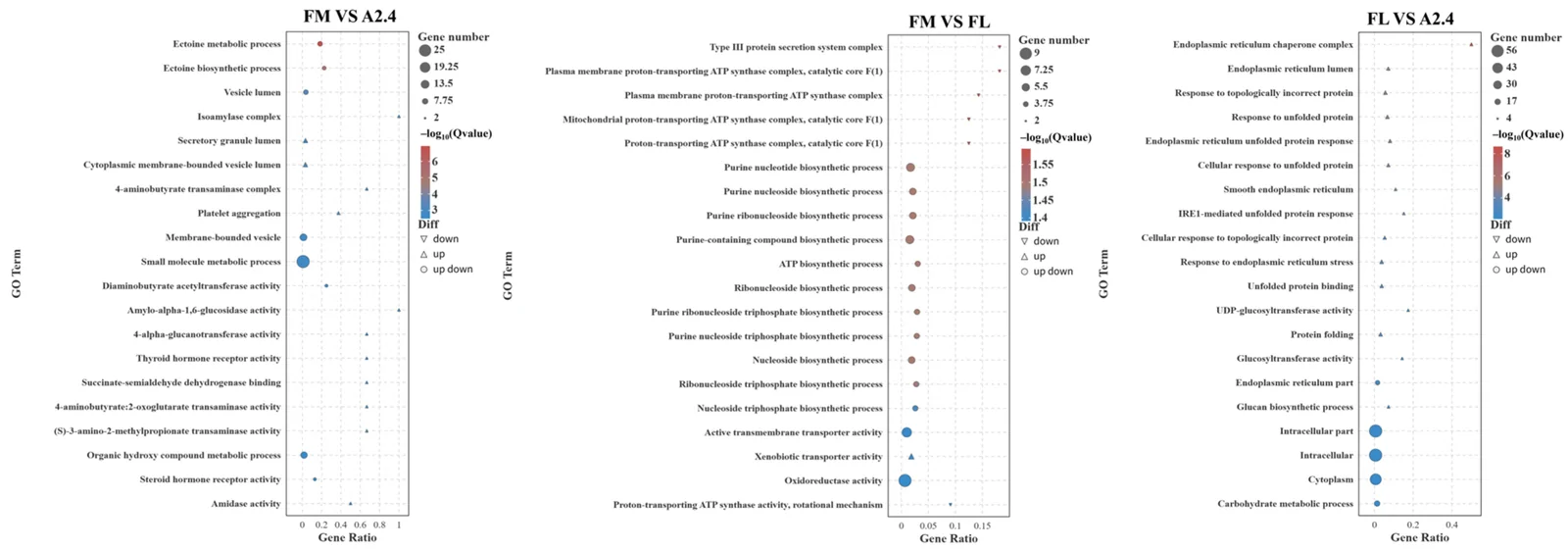
GO enrichment analysis of DEGs. DEGs were identified using EdgeR analysis based on three biological replicates per treatment group (*n* = 3).

GO enrichment analysis revealed dietary treatment-associated changes in functional categories in the shrimp hepatopancreas (Figure 8). Compared with the FM group, the upregulated DEGs in the A2.4 group were enriched in GO terms associated with secretion, including secretory granule lumen and cytoplasmic membrane-bounded vesicle lumen; carbohydrate metabolism, including isoamylase complex and amylo-alpha-1,6-glucosidase activity; and γ-aminobutyric acid metabolism, including 4-aminobutyrate transaminase complex and 4-aminobutyrate:2-oxoglutarate transaminase activity. In the FM vs. FL comparison, DEGs downregulated in the FL group were enriched in several terms related to proton-transporting ATP synthase, including mitochondrial proton-transporting ATP synthase complex, catalytic core F(1). In addition, multiple biosynthetic terms associated with purines, nucleosides, nucleotides and nucleoside triphosphates contained both upregulated and downregulated genes. In the FL vs. A2.4 comparison, most enriched GO terms were associated with upregulated genes. Relative to the FL group, the upregulated DEGs in the A2.4 group were predominantly enriched in functional categories related to the endoplasmic reticulum chaperone complex, endoplasmic reticulum lumen and protein folding.

**Figure 9:**
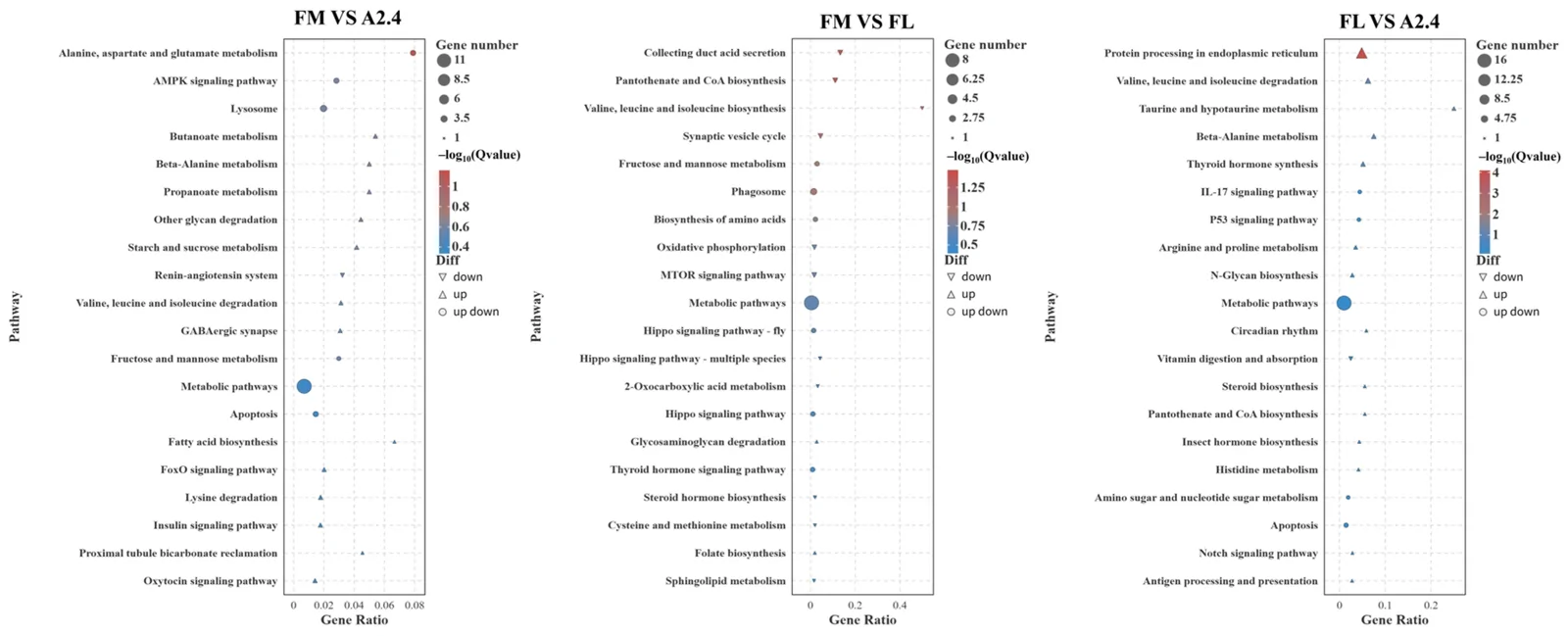
Bubble plot of KEGG pathway enrichment analysis. DEGs were identified using EdgeR analysis based on three biological replicates per treatment group (*n* = 3).

Based on the detected DEGs, pathway-level alterations were summarized using KEGG enrichment results and visualized by the GeneRatio distribution (Figure 9). In the FM vs. A2.4 comparison, most pathway-annotated DEGs were upregulated. Genes upregulated in the A2.4 group were primarily enriched in pathways related to amino acid metabolism (lysine degradation, β-alanine metabolism), organic acid metabolism (butanoate metabolism, propanoate metabolism), and carbohydrate degradation (other glycan degradation, starch and sucrose metabolism). The genes involved in these pathways primarily included *ABAT*, *gta-1*, *Fasn*, and *PCK2*. In the FM vs. FL comparison, DEGs downregulated in the FL group were primarily enriched in pathways including collecting duct acid secretion, pantothenate and CoA biosynthesis, mTOR signalling pathway, and oxidative phosphorylation. The genes primarily involved were *Vha55* and *ATP6V1B*. In addition, the fructose and mannose metabolism, phagosome, and biosynthesis of amino acids pathways contained both upregulated and downregulated DEGs. Compared with the FL group, DEGs upregulated in the A2.4 group were primarily enriched in pathways related to endoplasmic reticulum protein processing (protein processing in the endoplasmic reticulum), branched-chain amino acid degradation (valine, leucine and isoleucine degradation), sulfur-containing amino acid metabolism (taurine and hypotaurine metabolism), and β-alanine metabolism (β-alanine metabolism). The genes primarily involved included *CANX*, *ALDH1A1*, *Hsp90b1*, and *Hspa4l*.

#### 3.5.4. Correlation Analysis

**Figure 10:**
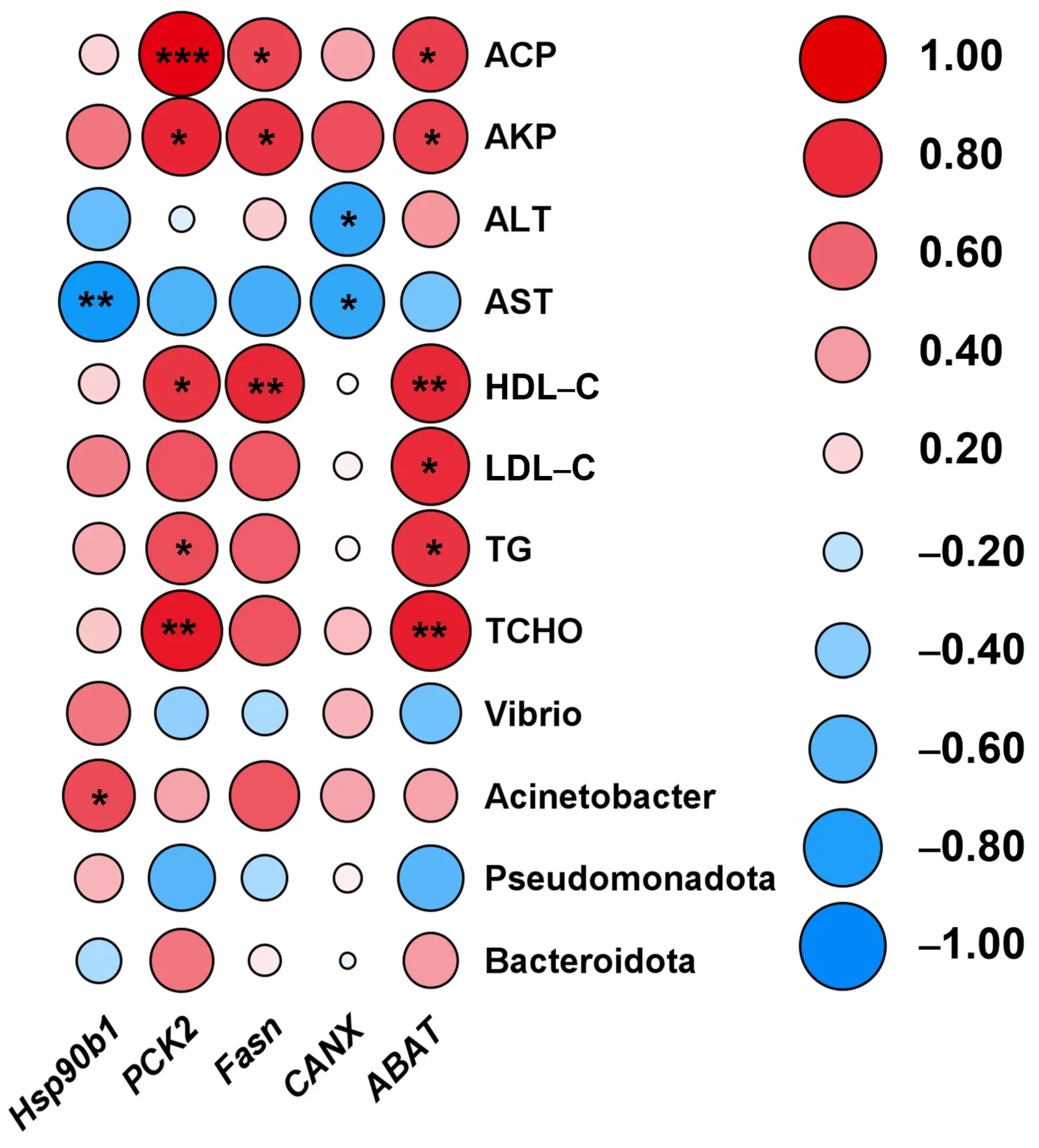
Correlation analysis of key genes with serum biochemical parameters and relative abundance of microbial taxa. The analysis was performed using three biological replicates from each of the FM, A2.4, and FL groups (*n* = 3). Transcriptomic expression data, serum biochemical parameters, and microbial abundance data from the corresponding biological replicates within each treatment group were used for Pearson correlation analysis. In each cell, the color of the circle represents the Pearson correlation coefficient (r) value, with red indicating a positive correlation and blue indicating a negative correlation. The size of the circle is proportional to the absolute value of the correlation coefficient (|r|); a larger circle indicates a stronger correlation. The statistical significance of the correlations is denoted by asterisks: * represents *p* < 0.05, **represents *p* < 0.01, and *** represents *p* < 0.001. Empty cells indicate no statistical significance (*p* ≥ 0.05). Due to the exploratory nature of this analysis and the limited number of biological replicates, these correlations not suitable for strict statistical interpretation.

An exploratory Pearson correlation analysis was conducted using data from three biological replicates in each of the FM, A2.4, and FL groups to assess the potential relationships between the expression levels of key genes and both serum biochemical parameters and microbial variables (Figure 10). PCK2 showed positive correlations with ACP, AKP, and lipid metabolism-related parameters, including HDL-C, LDL-C, TG, and T-CHO. Similar correlation patterns were observed for *Fasn*, which was positively associated with ACP, AKP, and the same lipid-related parameters. *Hsp90b1* expression exhibited negative correlations with serum AST and ALT activities. In addition, the relative abundance of Acinetobacter was positively correlated with the expression levels of *Hsp90b1*, *PCK2*, and *Fasn*. Significant correlations were also observed between *CANX* and *ABAT* expression and several measured variables. These results suggest potential associations between BmKn2-7-responsive genes and parameters related to hepatopancreatic function and lipid metabolism; however, these correlations should be interpreted cautiously due to the limited number of biological replicates and the exploratory nature of the analysis.

#### 3.5.5. Validation of Transcriptome by Quantitative Real-Time Polymerase Chain Reaction (qRT-PCR)

**Figure 11:**
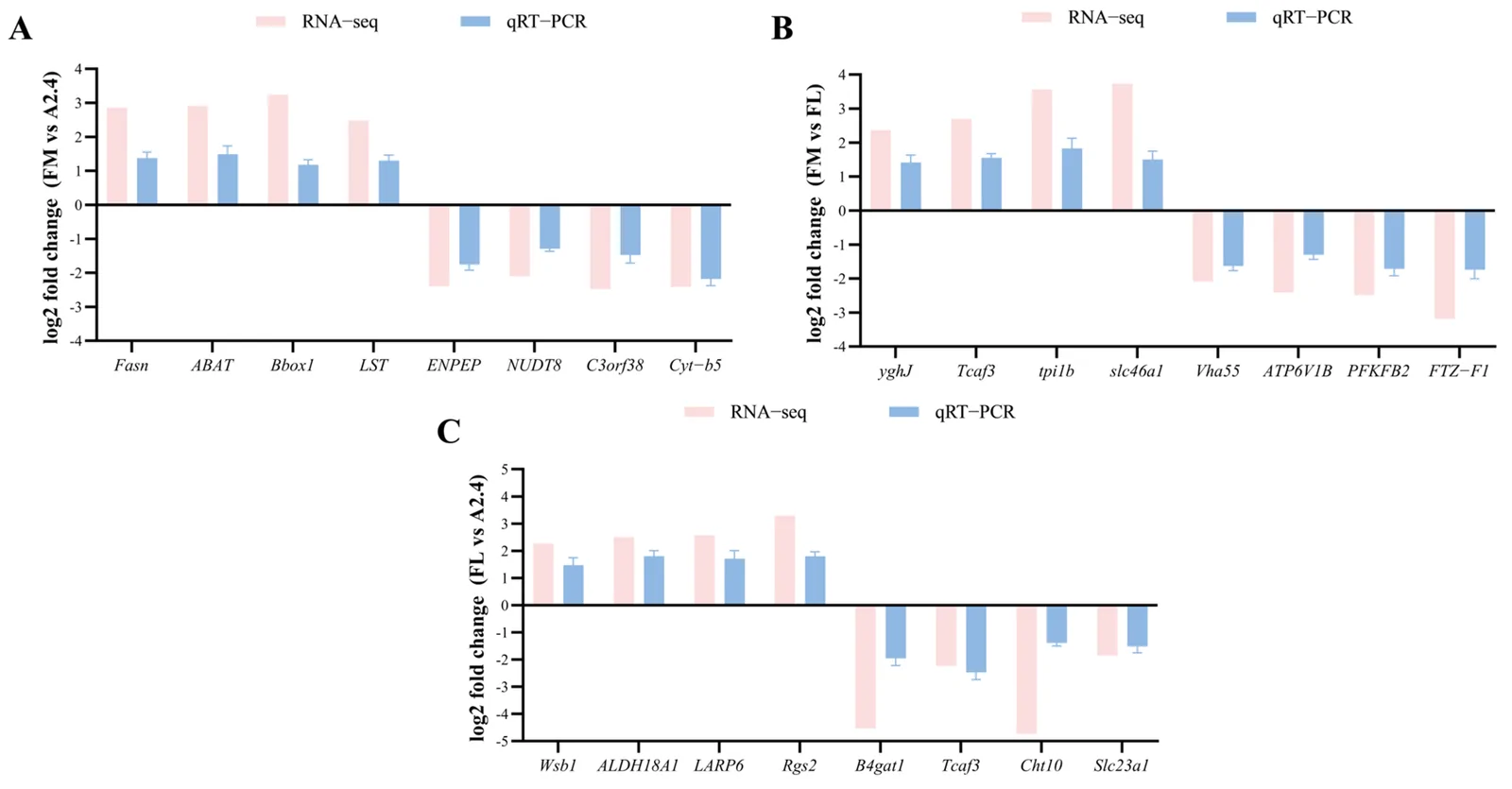
Comparison of the expression of RNA-Seq and qRT-PCR verification. (**A**): FM vs A2.4; (**B**): FL vs. FM; (**C**): FL vs A2.4.

Subsequently, 24 DEGs were selected for qRT-PCR validation to verify the expression patterns obtained from RNA-seq analysis. As shown in Figure 11, consistent expression trends identified across both RNA-seq and qRT-PCR analyses further corroborated the robustness of transcriptomic data.

### 3.6. Challenge Test Against V. parahaemolyticus

**Figure 12:**
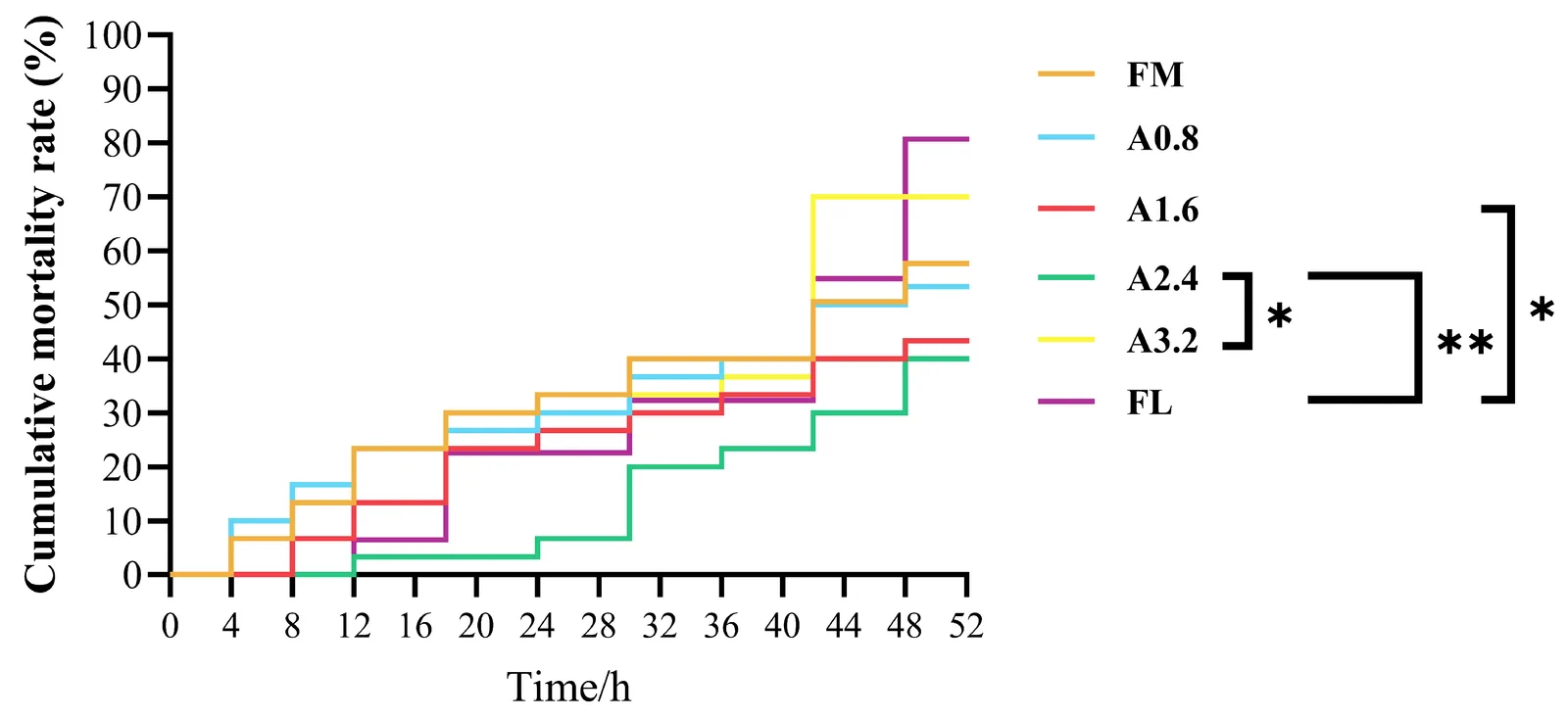
Effects of BmKn2-7 on cumulative mortality rate after injection with *V. parahaemolyticus* for *L. vannamei*. * *p* < 0.05, ** *p* < 0.01.

Cumulative mortality rates of shrimp following challenge with *V. parahaemolyticus* are presented in Figure 12. Following challenge, cumulative mortality was highest in the FL group, significantly exceeding that in the A1.6 (*p* < 0.05) and A2.4 (*p* < 0.01) groups. In contrast, the A2.4 group showed the lowest cumulative mortality, which was significantly lower than that of the A3.2 (*p* < 0.05) and FL (*p* < 0.01) groups. The cumulative mortality displayed a trend that initially decreased and subsequently increased among BmKn2-7 groups.

## 4. Discussion

The intensification of *L. vannamei* aquaculture, driven by escalating global demand, faces a critical constraint in the form of recurrent disease outbreaks [31,32]. Although conventional antibiotics remain effective for short-term disease control, their extensive use raises concerns regarding shrimp health, food safety, environmental impacts, and the dissemination of antimicrobial resistance, highlighting the need for sustainable alternatives [33]. A pharmacokinetic study showed that florfenicol was rapidly absorbed and distributed in *L. vannamei*, with the highest concentrations detected in the hepatopancreas. However, following a single oral administration of 10 mg/kg body weight, plasma concentrations remained below the MIC of *V. parahaemolyticus*, suggesting limited antimicrobial efficacy under these conditions [34]. Another study demonstrated that prolonged florfenicol administration induced intestinal damage and significantly altered the microbial communities and antibiotic resistance gene profiles in *L. vannamei*, although intestinal structure gradually recovered after drug withdrawal [29]. AMPs, characterized by broad-spectrum antimicrobial activity, diverse mechanisms of action, and relatively favorable environmental compatibility, have emerged as potential biocontrol agents for reducing antibiotic dependence in aquaculture [35]. Among AMPs, synthetic scorpion venom-derived peptides, such as BmKn2-7, possess distinctive structural features and antimicrobial properties that warrant further investigation [36]. However, despite increasing evidence that dietary AMPs, including scorpion venom-derived peptides and fermented soybean-derived peptides, can improve shrimp health and disease resistance, the effects of BmKn2-7 under practical dietary conditions in shrimp aquaculture remain unclear. Therefore, this study conducted an eight-week feeding trial to evaluate the effects of dietary BmKn2-7 supplementation on growth performance, physiological responses, intestinal microbiota, and hepatopancreatic transcriptomic profiles in *L. vannamei*, with florfenicol included as an antibiotic control. A limitation of this study is that the stability and recovery of BmKn2-7 after feed processing and storage were not directly measured. Future investigations should quantify peptide integrity in formulated diets to determine the actual bioavailable dosage.

In this study, supplementation with BmKn2-7 and florfenicol did not affect the growth performance of *L. vannamei*, including FBW, WGR, SGR, FCR, and SR. Similar results were observed in the study by Eslamloo et al., where dietary supplementation of 100–1600 mg AMPs did not significantly affect the growth performance of *Acipenser baeri* [37]. Importantly, BmKn2-7 supplementation across the tested dietary levels did not negatively affect these production-related parameters, supporting its potential suitability as a feed additive. However, differences were observed in morphometric indices, with CF and HSI values in the FL group being significantly lower than those in the other groups. The lower CF value may indicate alterations in body condition under florfenicol exposure, although the underlying changes in nutrient allocation require further investigation [38]. The reduced HSI observed in the FL group may reflect altered hepatopancreatic status, given the essential roles of the hepatopancreas in digestion, metabolism, and detoxification in shrimp [39]. Ren et al. reported that exposure to florfenicol at 200 mg/kg body weight for 6 days impaired hepatopancreatic function in *L. vannamei* [40]. This finding is significant, as it suggests that while florfenicol administration did not impede overall growth rates in this specific trial, it was associated with measurable physiological alterations manifesting as reduced CF and HSI, effects conspicuously absent in shrimp receiving BmKn2-7 supplementation. The absence of growth impairment in BmKn2-7-treated shrimp provides a basis for interpreting subsequent metabolic and molecular responses as effects associated with dietary peptide supplementation rather than secondary changes caused by altered growth performance.

Dietary BmKn2-7 supplementation induced clear dose-dependent changes in whole-body nutrient deposition, revealing its potential influence on metabolic regulation. Although moisture and crude protein contents showed minimal variation among treatments, the most prominent response was the increased crude lipid deposition at moderate BmKn2-7 levels (1.6–2.4 mg/kg). Similar increases in lipid deposition have been reported in other AMP-supplemented aquatic species and may reflect alterations in nutrient utilization or energy allocation [41]. This phenotype was consistent with transcriptomic evidence of altered lipid metabolic processes, including upregulation of Fasn and other AMPK-associated metabolic genes in the A2.4 group. In contrast, florfenicol markedly reduced lipid deposition, reinforcing its disruptive effects on metabolic homeostasis. Collectively, these nutrient deposition patterns suggest that BmKn2-7 may influence lipid metabolism beyond its direct antimicrobial activity and provide physiological context for the subsequent biochemical and transcriptional responses observed in this study.

Serum biochemical parameters provide valuable information regarding physiological and metabolic responses to dietary treatments [42]. Our results revealed changes in serum enzyme activities associated with metabolism and hepatopancreatic function, together with alterations in lipid-related parameters. Among the tested concentrations, dietary BmKn2-7 at 2.4 mg/kg (A2.4) produced the most pronounced physiological responses. The enzymatic activities of ACP and AKP in shrimp serum play crucial roles in immune defense mechanisms and metabolic processes [43,44]. This dose significantly stimulated phosphatase activities (ACP and AKP), indicative of enhanced metabolic or immune-related processes. The increase in ACP activity was partially attenuated at the higher 3.2 mg/kg dose, further suggesting a concentration-dependent response with maximal effects observed at intermediate BmKn2-7 levels (1.6–2.4 mg/kg). Similarly, Gyan et al. reported that dietary AMP supplementation at 0.2–0.4% increased ACP and AKP activities in shrimp, whereas a higher inclusion level (0.6%) reduced these enzyme activities [19]. Although ACP and AKP activities provided evidence of altered innate immune-related responses following BmKn2-7 supplementation, a more comprehensive understanding of its immunomodulatory effects requires further evaluation of additional immune parameters and immune-related gene expression. Meanwhile, serum lipid profiles showed that A2.4 increased HDL-C, LDL-C, T-CHO, and TG levels, suggesting alterations in lipid transport and hepatopancreatic metabolic status [45]. Aminotransferase activities were further evaluated as indicators of hepatopancreatic stress. The marked elevation of ALT activity in the FL group compared with all other treatments suggested alterations in hepatopancreatic physiological status associated with florfenicol exposure [46]. The hepatic injury observed in the FL group may be attributed to metabolic oxidative stress and mitochondrial dysfunction [47]. Although AST activity showed a more complex response pattern, the higher AST level in the FL group than in the A2.4 group further indicated that florfenicol exposure was associated with changes in hepatopancreatic-related biochemical responses. Notably, ALT activities in shrimp receiving BmKn2-7 at 0.8–2.4 mg/kg remained comparable to those in the FM group, suggesting that BmKn2-7 supplementation did not cause detectable alterations in this hepatopancreatic biochemical indicator under the present experimental conditions. The contrasting responses between BmKn2-7 and florfenicol treatments suggest that BmKn2-7 supplementation was associated with a relatively stable hepatopancreatic biochemical profile, whereas florfenicol treatment resulted in more pronounced alterations in hepatopancreatic-related indicators. Overall, dietary BmKn2-7 at 2.4 mg/kg increased serum phosphatase activities and modified lipid-related parameters without apparent adverse effects on hepatopancreatic status, whereas florfenicol exposure was associated with elevated ALT activity. The partial attenuation of benefits at 3.2 mg/kg underscores the critical importance of dose optimization for BmKn2-7 application.

Intestinal microbiota analysis revealed distinct changes in microbial community composition in response to BmKn2-7 and florfenicol (FL) supplementation. While FL, as expected for a broad-spectrum antibiotic, was associated with marked alterations in microbial community structure, including reduced diversity and changes in the relative abundance of taxa such as *Tenacibaculum* and unclassified bacteria, BmKn2-7 supplementation at 2.4 mg/kg was associated with a more balanced and diverse microbial community. The Shannon and Simpson indices were numerically highest in the A2.4 group, although no significant pairwise differences were detected after Dunn’s post hoc test with Bonferroni correction, suggesting a trend toward higher microbial diversity at this dietary level. Similarly, dietary supplementation with APSH-07 was reported to increase gut microbial richness and diversity in *Larimichthys crocea*, consistent with the present findings [41]. Together, these findings suggest that dietary BmKn2-7 supplementation was associated with a more balanced intestinal microbial profile compared with the broad microbiota disturbance observed in the FL group. The most notable microbiota-associated change in the A2.4 group was the lower relative abundance of potential pathogenic *Vibrio* taxa accompanied by an enrichment of *Acinetobacter*. The reduction in relative abundance of *Vibrio* observed after BmKn2-7 supplementation may be associated with the antimicrobial properties of BmKn2-7 reported previously; however, the contribution of direct antibacterial activity versus microbiota-mediated effects requires further investigation. Interestingly, the effect of BmKn2-7 supplementation on *Vibrio* abundance appeared to be non-linear. While the A2.4 treatment markedly reduced *Vibrio* abundance compared with FM, the higher supplementation level (A3.2) resulted in an increased *Vibrio* proportion, suggesting that excessive supplementation may alter microbial interactions and potentially favor opportunistic bacterial proliferation. Further studies are needed to elucidate the underlying mechanisms. Some *Acinetobacter* groups may contribute to enhancing the immune capacity and disease resistance of aquatic species [48]; however, this genus also includes opportunistic pathogens, and its potential beneficial effects require further validation. This altered microbial profile, together with increased alpha diversity, suggests that BmKn2-7 supplementation may be associated with changes in pathogen-associated taxa and microbial community stability. This microbial profile was consistent with the enhanced disease resistance observed in the A2.4 group. In contrast, the FL group exhibited a distinct microbial community characterized by lower *Vibrio* abundance, increased representation of Demequina and *Tenacibaculum*, and a higher proportion of unclassified taxa. This structural disruption, consistent with FL’s known antibiotic impact on microbiota, was further evidenced by significant β-diversity separation from other groups [46]. Antibiotic-induced alterations in the intestinal microbiota may result from the suppression of susceptible microorganisms and the proliferation of opportunistic bacteria [49]. Co-occurrence network analysis further indicated differences in microbial association patterns between FL and BmKn2-7 treatments. Mantel analysis indicated that microbiota composition was correlated with HSI and ALT, consistent with the changes in hepatopancreatic-related parameters observed after florfenicol exposure. These findings suggest a potential association between gut microbiota alterations and hepatopancreatic responses following dietary treatments. The BmKn2-7-associated microbiota profile may be related to the maintenance of hepatopancreatic physiological status, whereas florfenicol-associated microbial alterations may be associated with changes in hepatopancreatic function [50].

The hepatopancreatic transcriptome revealed distinct regulatory responses to dietary BmKn2-7 and florfenicol supplementation. GO and KEGG analyses showed that most pathways associated with the DEGs in the A2.4 group were upregulated. GO analysis revealed that, compared with the FM group, BmKn2-7 treatment upregulated a subset of genes in the shrimp hepatopancreas associated with carbohydrate metabolism, γ-aminobutyric acid-related processes, vesicular transport, and secretion. These findings suggest that BmKn2-7 may enhance nutrient substrate utilization and energy metabolism while promoting the secretory function of the hepatopancreas. The altered lysine degradation pathway suggests changes in microbial amino acid utilization, which may potentially influence lysine turnover and availability to the host. β-alanine is a precursor for the synthesis of vitamin B5, which is a component of intracellular coenzyme A and acyl carrier proteins [51]. Butyrate and propionate can participate in energy metabolism and contribute to the maintenance of intestinal homeostasis [52]. Changes in butanoate and propanoate metabolism further suggest that A2.4 may influence microbial short-chain fatty acid-related metabolic potential. Nevertheless, these KEGG results represent microbial functional potential rather than direct measurements of metabolites or host metabolic responses.

In contrast, compared with the FL group, the A2.4 group showed upregulation of genes associated with the endoplasmic reticulum and protein folding. These findings suggest that the A2.4 group may exhibit more active endoplasmic reticulum-mediated protein quality control and proteostasis regulation. In addition, multiple terms associated with proton-transporting ATP synthase were predominantly driven by genes downregulated in the FL group. Genes associated with the catalytic core of mitochondrial proton-transporting ATP synthase F1 were downregulated in the FL group, suggesting potential alterations in mitochondrial energy metabolism and ATP synthesis-related processes. Previous cellular studies have suggested that florfenicol may affect mitochondrial function, including mitochondrial protein translation, membrane potential maintenance, ATP production, and reactive oxygen species homeostasis [53]. These findings provide mechanistic support for the downregulation of ATP synthase-related genes observed in the present study. Compared with the FM group, BmKn2-7 supplementation was associated with transcriptional changes in pathways related to amino acid metabolism, organic acid metabolism, and carbohydrate degradation in the shrimp hepatopancreas. BmKn2-7 supplementation may influence nutrient utilization and metabolic processes in the hepatopancreas. Moreover, transcriptomic analysis showed that the expression levels of *ABAT*, *gta-1*, *Fasn*, and *PCK2* were highest in the A2.4 group. ABAT encodes 4-aminobutyrate aminotransferase, an enzyme that catalyzes the conversion of γ-aminobutyric acid (GABA) to succinic semialdehyde [54]. Given that *gta-1* is annotated as a mitochondrial 4-aminobutyrate aminotransferase, the concomitant upregulation of *ABAT* and *gta-1* suggests that BmKn2-7 may enhance GABA catabolism and GABA shunt-associated metabolic activity. This is consistent with the enrichment of 4-aminobutyrate aminotransferase-related terms in the GO analysis and of butanoate and β-alanine metabolism pathways in the KEGG analysis. PCK2 catalyzes the mitochondrial conversion of oxaloacetate to phosphoenolpyruvate and serves as a key metabolic node linking tricarboxylic acid cycle intermediates to gluconeogenesis and carbon-skeleton redistribution [55]. Fatty acid synthase (FASN) is a key enzyme responsible for the de novo synthesis of long-chain fatty acids from acetyl-CoA and malonyl-CoA [56]. The elevated expression of PCK2 and Fasn in the A2.4 group likely reflects enhanced nutrient conversion and energy supply induced by BmKn2-7. Compared with the FM group, florfenicol exposure was associated with transcriptional alterations in pathways related to coenzyme A metabolism, amino acid metabolism, and nutrient-sensing signaling in the shrimp hepatopancreas. The differences in proton-transporting ATPase-related functions between the FM and FL groups were primarily associated with *Vha55* and *ATP6V1B*. Vha55 has been identified as a gene encoding the B subunit of vacuolar-type H^+^-ATPase (V-ATPase), while ATP6V1B likewise encodes the B subunit of the cytosolic V1 domain of V-ATPase [57]. Therefore, the concomitant downregulation of *Vha55* and *ATP6V1B* in the FL group suggests that florfenicol may impair V-ATPase-mediated proton transport in the hepatopancreas. Compared with the FL group, the A2.4 group exhibited a transcriptional signature characterized by the upregulation of genes involved in endoplasmic reticulum protein processing, accompanied by enhanced N-glycan biosynthesis. The enrichment of the protein processing in endoplasmic reticulum pathway was primarily driven by molecular chaperone-related genes, including *CANX*, *Hsp90b1*, and *Hspa4l*. These findings are consistent with enhanced endoplasmic reticulum-associated protein quality control and proteostasis regulation in the A2.4 group. In addition, enrichment of amino acid metabolism-related pathways suggests that BmKn2-7 supplementation may influence amino acid turnover and utilization in the hepatopancreas. Although transcriptomic analysis was performed using the optimal BmKn2-7 supplementation group, future studies including additional dosage groups (e.g., A3.2) are required to further elucidate dose-dependent molecular responses.

The challenge test further confirmed BmKn2-7’s functional efficacy in enhancing disease resistance, while exposing potential limitations of florfenicol. Previous studies showed that BmKn2-7 exhibited direct antibacterial activity against *V. parahaemolyticus*, with a minimum inhibitory concentration (MIC) of 125 μg/mL, and that its antibacterial action was associated with membrane disruption. Supplementation with BmKn2-7 at 2.4 mg/kg resulted in the lowest cumulative mortality, which was significantly lower than that of the FL group (*p* < 0.01) and the A3.2 group (*p* < 0.05). This identifies 2.4 mg/kg as the apparently optimal dose for maximal protection—a finding aligning precisely with its superior performance in earlier metrics of immune readiness and hepatopancreatic health. Consistently, previous studies have shown that dietary AMPs enhance resistance against *Vibrio* infection in *L. vannamei*, likely through combined bactericidal and immunomodulatory actions [19,58,59]. In contrast, the FL group exhibited the highest cumulative mortality, which was significantly higher than that of the A1.6 (*p* < 0.05) and A2.4 (*p* < 0.01) groups. This outcome was consistent with the altered hepatopancreatic status and intestinal microbiota composition observed in florfenicol-treated shrimp, which may have contributed to reduced resilience during acute *Vibrio* challenge [60]. The non-linear mortality pattern among BmKn2-7 treatments, characterized by decreased mortality from A0.8 to A2.4 followed by an increase at A3.2, suggests a concentration-dependent response. The lower efficacy at A0.8 may reflect insufficient biological stimulation, whereas the reduced protection at A3.2 may be associated with altered metabolic responses or host–peptide interactions. Comparable dose-dependent effects have been reported for other bioactive proteins, such as high-dose lactoferrin exerting adverse effects in *Dicentrarchus labrax* [61]. Although the intramuscular injection challenge provides a consistent assessment of systemic resistance against *V. parahaemolyticus*, it does not fully represent the natural infection process. This challenge model bypasses the initial barriers of infection, including the intestinal microbiota and gut barrier, and therefore cannot fully evaluate their contribution during pathogen invasion. Collectively, these results indicate that BmKn2-7 supplementation at 2.4 mg/kg improved resistance to *V. parahaemolyticus* challenge under the present experimental conditions, whereas florfenicol treatment was associated with reduced survival and altered physiological status. Although BmKn2-7 supplementation did not cause detectable adverse physiological effects under the conditions tested, further studies evaluating tissue residue, distribution, and elimination kinetics are required to comprehensively assess its food safety before practical application.

## 5. Conclusions

This study indicates that dietary supplementation with the antimicrobial peptide BmKn2-7 at 2.4 mg/kg represents a viable alternative to the antibiotic florfenicol in shrimp aquaculture under the present experimental conditions. BmKn2-7 supplementation improved resistance to *V. parahaemolyticus* challenge without negatively affecting growth performance, potentially through coordinated modulation of physiological, microbial, and metabolic processes distinct from florfenicol treatment. Rather than inducing adverse effects, dietary BmKn2-7 supplementation was associated with changes in innate immune-related enzyme activities, metabolic parameters, and intestinal microbial community structure, suggesting its potential role in maintaining physiological homeostasis in *L. vannamei*. Transcriptomic analyses showed that BmKn2-7 promoted hepatopancreatic metabolic remodeling, particularly in amino acid, organic acid, and carbohydrate metabolism. BmKn2-7 supplementation was also associated with enrichment of endoplasmic reticulum protein processing pathways and increased expression of genes including *ABAT*, *gta-1*, *PCK2*, *CANX*, *Hsp90b1*, and *Hspa4l*. In contrast, florfenicol reduced *Vha55* and *ATP6V1B* expression, suggesting impaired V-ATPase-mediated proton transport and cellular homeostatic functions. Therefore, BmKn2-7 shows promising potential to improve host metabolic homeostasis, support hepatopancreatic function, and enhance overall health resilience. This work provides a potentially sustainable approach for disease management in aquaculture, with the potential to contribute to mitigating antibiotic overuse.

## Figures and Tables

**Table 1:** Ingredient compositions and nutrient contents of test diets (%, air dry matter).

Ingredients	Diets					
	FM	A0.8	A1.6	A2.4	A3.2	FL
Fish Meal	25	25	25	25	25	25
Soybean Meal	11	11	11	11	11	11
Peanut Meal	11	11	11	11	11	11
Corn Gluten Meal	13	13	13	13	13	13
Shrimp Shell Meal	5	5	5	5	5	5
Wheat Flour	25	25	25	25	25	25
Fish Oil	2	2	2	2	2	2
Soy Lecithin	2.5	2.5	2.5	2.5	2.5	2.5
Monocalcium Phosphate	2	2	2	2	2	2
Vitamin and mineral premix ^a^	1	1	1	1	1	1
Attractant	0.1	0.1	0.1	0.1	0.1	0.1
Vitamin C	0.1	0.1	0.1	0.1	0.1	0.1
Antioxidant	0.05	0.05	0.05	0.05	0.05	0.05
Choline Chloride	0.5	0.5	0.5	0.5	0.5	0.5
Microcrystalline Cellulose	1.75	1.75	1.75	1.75	1.75	1.75
BmKn2-7 (mg/kg)	0	0.8	1.6	2.4	3.2	0
Florfenicol (mg/kg)	0	0	0	0	0	3.2
Nutrient contents (air dry matter) ^b^						
Moisture	7.81	7.70	7.67	7.73	7.60	7.89
Crude protein	41.10	40.76	40.85	40.57	40.82	40.57
Crude lipid	6.65	6.54	6.66	6.60	6.72	6.32
Ash	10.17	10.16	10	10.21	10.17	9.97

Note: a: Premix offered by Qingdao Master Biotech Co., Ltd. Vitamin and Mineral Premix (/Kg) includes the following: vitamin A, 500,000 IU; vitamin D3, 100,000 IU; vitamin E, 4000 mg; vitamin K3, 1000 mg; thiamine, 500 mg; riboflavin, 1000 mg; pyridoxine, 1000 mg; cobalamin, 2 mg; niacin, 4000 mg; calcium pantothenate, 2000 mg; folic acid, 100 mg; vitamin C, 15,000 mg; iron, 10,000 mg; copper, 300 mg; zinc, 5000 mg; manganese, 1200 mg; iodine, 80 mg; selenium, 30 mg; cobalt, 20 mg. b: Crude protein, crude lipid, moisture and ash were all the observed values.

**Table 2:** Primers used for qRT-PCR validation of selected differentially expressed genes in *L. vannamei*.

Primer Name	Sequence (5′-3′) F	Sequence (5′-3′) R
β-actin	CTATGTCGCCTTGGACTTCG	TGTTGTAGGTGGTCTCGTGGA
*ENPEP*	TGGAATTGGGTGAGGTCGAA	TCCCATACAGGGTGGGATCA
*NUDT8*	ACGACGTGTGGTTCCGATAC	TCCTCTGGGCATCTTGCTCT
*C3orf38*	CTTCGCTCAACAAGAAGGCG	TGCAGCCATTATGCCTTTGC
*Cyt-b5*	CTCGGGAGATGATGGCAGAC	CTTGACTGTGGCAGGGCATA
*Fasn*	CAAACAACACTGCTCTGGGC	GGGCGTCCTTCAGGATCATT
*ABAT*	AGAAGTGACCACCTCAGGCTA	AGATGAGGGCAGGACGAAGA
*Bbox1*	AGGATCTCCCCAAAGGTCCC	AGTCGATGAGTGGCACTTGG
*LST*	CAAATGCCCCAGATGAAGGC	TGGGATGTGCGAAGTCCTTA
*Vha55*	CTGGAATCCGACTCGCAAGA	CCTGACGACAAATCTGGGCT
*ATP6V1B*	AGGTGCCAGGTAGAAGAGGT	CCCGTCAGATCAGGGATTGG
*PFKFB2*	ACGGTCGAGTGTTCGTTGTT	AGCGTTGACAGAGGAACCTG
*FTZ-F1*	ACTGTCACAGGCAAGACTCG	ATAGTGGTAGCCCGACACCT
*yghJ*	TGGCTACAGCGGAAGATTGC	GCCTTCACGACGTTTGTCAC
*Tcaf3*	AAGCGACTCCGAAACAGCAT	ATCCCTCGCGAGATCGACTA
*tpi1b*	TCGAGGACCAGCAGGGATAA	TGCAGGGAATGTGAGTGGAC
*slc46a1*	AGTCGAAGACCAACACGACC	GAAGGAGAGGACGATCAGCG
*B4gat1*	GCCATCCCCTTCCACAAGAA	TGCAGCTTCTTGATCCCTGG
*Cht10*	TCCTGCCAGAACGGACTCTA	GAGCCGTCCAACATAGCGTA
*Slc23a1*	ACTCTGGAACGTCAGAGAGGA	GCCGACCATCGTCAGGTAAT
*Wsb1*	CTGCCGTGGAACCGATACAA	CTCGCGTATCACCAGATGCT
*ALDH18A1*	ACTTCTGGAAACGAGGCAGG	TTTTAGAGAGCCTTGGCCCG
*LARP6*	CTTTACCAGCACGCAGAGGA	GGTTGGGCGAGGTATCGTAG
*Rgs2*	CCGTTCAACTCCTTCGGTGA	TGAACGTCGGGGAGAGTTTG

Note: Primers were designed based on the transcriptome sequencing sequences.

**Table 3:** Effects of BmKn2-7 on the growth performance in *L. vannamei*.

Parameter	Group					
	FM	A0.8	A1.6	A2.4	A3.2	FL
IBW	0.26 ± 0.00 ^a^	0.26 ± 0.00 ^a^	0.26 ± 0.00 ^a^	0.26 ± 0.00 ^a^	0.26 ± 0.00 ^a^	0.26 ± 0.00 ^a^
FBW	13.71 ± 0.45 ^a^	13.71 ± 0.13 ^a^	13.42 ± 0.43 ^a^	13.49 ± 0.25 ^a^	13.97 ± 0.23 ^a^	13.87 ± 0.09 ^a^
WGR (%)	4907.76 ± 149.51 ^a^	5002.44 ± 39.96 ^a^	4764.54 ± 82.26 ^a^	4835.36 ± 16.67 ^a^	4956.06 ± 117.61 ^a^	4951.55 ± 75.58 ^a^
SGR (% day^−1^)	6.98 ± 0.05 ^a^	7.02 ± 0.01 ^a^	6.93 ± 0.03 ^a^	6.93 ± 0.00 ^a^	7.00 ± 0.04 ^a^	7.00 ± 0.02 ^a^
FCR	1.04 ± 0.03 ^a^	1.03 ± 0.01 ^a^	1.08 ± 0.02 ^a^	1.06 ± 0.00 ^a^	1.04 ± 0.02 ^a^	1.03 ± 0.00 ^a^
SR (%)	96.67 ± 0.83 ^a^	96.67 ± 0.83 ^a^	95.00 ± 2.88 ^a^	95.00 ± 0.00 ^a^	94.16 ± 1.66 ^a^	95.00 ± 1.44 ^a^
CF (%)	0.98 ± 0.04 ^a^	1.02 ± 0.09 ^ab^	0.96 ± 0.06 ^ab^	0.94 ± 0.03 ^ab^	1.00 ± 0.04 ^a^	0.82 ± 0.00 ^b^
HSI (%)	5.31 ± 0.40 ^a^	5.19 ± 0.29 ^a^	5.24 ± 0.21 ^a^	5.18 ± 0.25 ^a^	4.97 ± 0.37 ^a^	3.89 ± 0.23 ^b^

Note: Data are presented as mean ± SEM (*n* = 3), different letter superscripts indicate significant differences (*p* < 0.05). Growth performance was calculated using the following formula: weight gain rate (WGR, %) = (final body weight—initial body weight)/initial body weight × 100; specific growth rate (SGR, %.day−1) = (ln final weight—ln initial weight)/days of the feeding experiment × 100; feed conversion ratio (FCR) = feed consumed/weight gain; survival rate (SR, %) = (final number of shrimp/initial number of shrimp) × 100; condition factor (CF, %) = (Body Weight/Body Length^3^) × 100; Hepatosomatic Index (HSI, %) = (hepatopancreas Weight/Body Weight) × 100.

**Table 4:** Effects of BmKn2-7 on whole-body proximate composition of *L. vannamei*.

Index	Group					
	FM	A0.8	A1.6	A2.4	A3.2	FL
Moisture	73.40 ± 0.53 ^a^	73.33 ± 0.52 ^a^	73.74 ± 0.50 ^a^	73.74 ± 0.52 ^a^	72.95 ± 0.71 ^a^	73.30 ± 0.43 ^a^
Crude protein	72.94 ± 0.15 ^a^	72.91 ± 0.21 ^a^	72.63 ± 0.17 ^a^	71.55 ± 0.35 ^b^	72.86 ± 0.09 ^a^	72.60 ± 0.01 ^a^
Crude lipid	6.11 ± 0.02 ^d^	6.42 ± 0.12 ^c^	7.20 ± 0.69 ^a^	6.83 ± 0.16 ^b^	5.80 ± 0.02 ^e^	6.07 ± 0.00 ^de^
Crude ash	11.55 ± 0.15 ^c^	12.48 ± 0.02 ^a^	11.50 ± 0.02 ^c^	12.38 ± 0.12 ^ab^	12.19 ± 0.10 ^ab^	12.10 ± 0.02 ^b^

Note: Data are presented as mean ± SEM (*n* = 3), different letter superscripts indicate significant differences (*p* < 0.05). Moisture is expressed on a wet-weight basis, whereas crude protein, lipid, and ash are expressed on a dry-matter basis.

## Data Availability

The raw transcriptome sequencing data and gut microbiota sequencing data generated in this study have been deposited in the National Center for Biotechnology Information (NCBI) repository under BioProject accession numbers PRJNA1498765 and PRJNA1499116, respectively.

## Abbreviations

The following abbreviations are used in this manuscript:

IBW	initial body weight
FBW	final body weight
WGR	Weight gain rate
FCR	Feed conversion ratio
SGR	Specific growth rate
SR	Survival rate
CF	Condition Factor
HSI	Hepatosomatic Index
AKP	alkaline phosphatase
ACP	acid phosphatase
AST	aspartate aminotransferase
ALT	alanine aminotransferase
HDL-C	high-density lipoprotein
LDL-C	low-density lipoprotein
ENPEP	Glutamyl aminopeptidase
NUDT8	Nudix hydrolase 8
C3orf38	Chromosome 3 open reading frame 38
Cyt-b5	Cytochrome b5
Fasn	Fatty acid synthase
ABAT	4-Aminobutyrate aminotransferase
Bbox1	Gamma-butyrobetaine hydroxylase 1
LST	sulfotransferase
Vha55	vacuolar ATP synthase subunit B
ATP6V1B	ATPase H^+^ transporting V1 subunit B
PFKFB2	6-Phosphofructo-2-kinase
FTZ-F1	Ftz transcription factor 1
yghJ	TRPM8 channel-associated factor homolog
Tcaf3	TRPM8 channel-associated factor 3
tpi1b	Triosephosphate isomerase 1b
slc46a1	Solute carrier family 46 member 1
B4gat1	Beta-1,4-glucuronyltransferase 1
Cht10	Chitinase 10
Slc23a1	Solute carrier family 23 member 1
Wsb1	WD repeat and SOCS box containing 1
ALDH18A1	Aldehyde dehydrogenase 18 family member A1
LARP6	La ribonucleoprotein 6, translational regulator
Rgs2	Regulator of G protein signaling 2
